# Analytical prediction of groundwater loss in deep coal mines induced by ground vibration

**DOI:** 10.1038/s41598-025-05970-6

**Published:** 2025-07-22

**Authors:** Pieride Mabe Fogang, Bingjie Huo, Hervé Losaladjome Mboyo, Rong Hai, Songtao Zhang, Lesly Dasilva Wandji Djouonkep, Dieudonné Bisso

**Affiliations:** 1 School of Mining, Liaoning Technical University, Fuxin, 123000 China; 2 Department of Civil Engineering, National Advanced School of Public Works, Yaoundé, 510 Cameroon; 3 School of New Energy and Mining, Xinjiang University of Technology, Hetian, Xinjiang 848000 China; 4 Institute of Fine Organic Chemicals and Organic Materials, School of Chemistry and Chemical Engineering, Wuhan University of Science and Technology, Wuhan, 430081 China; 5 Department of Earth Sciences, University of Yaoundé I, Yaoundé, 812 Cameroon

**Keywords:** Coal mining, Groundwater dynamics, Predictive modeling, Aquifer permeability, Civil engineering, Engineering, Mathematics and computing

## Abstract

Deep coal mining induces geomechanical perturbations that threaten aquifer integrity. This study develops an analytical model coupling Fourier’s heat conduction and Cauchy’s momentum equations to predict groundwater depletion under dynamic stress from vibrations (0–6 MPa). Laboratory tests on Datong Mine samples (coal seam No. 12) yielded baseline parameters, including soil cohesion (*C* = 1.0 MPa) and Poisson ratio (*ν* = 0.35). The simulation uses an effective elastic modulus (*E* = 12.5 GPa) to represent the fractured coal-rock mass under vibrational loading. Results show vibration-induced fractures increase permeability by 15–25% initially, but subsequent compaction reduces it by 60%, with peak vertical displacements of 0.18 m. Vibrational loads exceeding a critical stress magnitude of 6 MPa exacerbate hydraulic conductivity variations**,** altering pore pressure distributions and threatening aquifer integrity. The model, validated via ABAQUS simulations, provides a scalable tool for mitigating water loss in mining environments. This research highlights the criticality of harmonizing geomechanical simulations with hydrogeological assessments to advance groundwater management strategies. The proposed analytical solution offers a scalable solution for mitigating environmental and operational risks across diverse mining geologies, ensuring resource sustainability and operational resilience against geohydrological instabilities.

## Introduction

Deep coal mining has been pivotal in powering global industrial development, but its ecological consequences are profound. Recent studies have linked mining activities to substantial impacts on groundwater resources, altering recharge, runoff, and drainage conditions. These changes often lead to the creation of water-conducting fracture zones that disrupt aquifer systems. The connection between mining depths and severe groundwater depletion in overlying aquifers observed drops of up to 80 m has been well-documented^1^. Additionally, coal mining has been shown to significantly alter hydrological cycles^2^. Furthermore, understanding fractures in overlying strata is crucial for effective water management^3^. As the demand for coal continues, developing strategies to mitigate these impacts while maintaining operational efficiency remains a pressing challenge. However, few studies have addressed the issue of groundwater loss under dynamic loads, particularly in relation to deep mining activities.

These recent studies have increasingly focused on modeling groundwater loss in mining-affected areas, especially through numerical, empirical, and analytical frameworks. Numerical models, such as those based on finite element and finite difference methods, have been widely employed to simulate groundwater flow around coal mines, integrating geomechanical deformation and hydrogeological responses^4,5^. Empirical models, often derived from field monitoring data, provide site-specific predictions of aquifer drawdown and seepage rates but lack general applicability^6^. Analytical models, while less common due to the complexity of coupled hydro-mechanical behavior in fractured rock masses, offer valuable insights for generalised scenarios and parameter sensitivity analyses^7^. However, these models typically consider steady-state or quasi-static loading conditions, and few have incorporated the transient, dynamic effects associated with coal mining-induced ground vibrations.

Soil vibrations induced by mining activities can lead to substantial changes in groundwater dynamics, exacerbating water loss through the creation of fractures and increased permeability in the surrounding rock. Established research highlights the role of these vibrations altering groundwater flow paths, causing aquitard fractures and ground subsidence^3,8–11^(A.1). For instance, studies indicate that the average distance of water infiltration through cracks can reach up to 0.11 m, with vibrations creating stress concentrations that facilitate water movement through geological formations. Furthermore, the interaction between ground vibrations and geological structures can result in complex hydrogeological responses, necessitating a more nuanced approach to water management in mining operations.

Ground vibrations from mining equipment and blasting operations induce dynamic stresses on subsurface structures. These vibrations create fractures that alter water flow paths, leading to significant water loss from aquifers. Hydraulic and mechanical loads can generate structural cracks, causing irregular subsidence and reducing water retention^12^. Ground subsidence has been observed to diminish aquifer connectivity, exacerbating water contamination risks^10^. Additionally, elastic wave propagation within fractured zones (A.1) has been reported to increase permeability, further enhancing water loss^3^. The complex potential analysis used to evaluate soil deformation around cylindrical cavities under vibratory conditions revealed substantial shifts in pore pressure and flow characteristics^13^. Compaction and seepage dynamics in coal and rock masses have also been linked to vibration intensity, indicating a direct correlation with water loss^3^. These findings highlight the critical need for predictive modeling to mitigate groundwater depletion during mining.

Previous studies have extensively analyzed the physical–mechanical properties of fractured rock strata. However, limitations persist in incorporating vibratory loads into groundwater loss predictions. This study aims to address these gaps by developing an analytical model based on Fourier’s law and Cauchy’s momentum equation. Laboratory tests measured soil cohesion (*C*), modulus of elasticity (*E*), and Poisson ratio (*v*) using samples from the Datong mines. The model predicts dynamic stress from vibration distributions and hydrogeological changes, providing novel insights into water retention strategies during mining operations. Expected results include improved accuracy in groundwater distribution predictions and reduced environmental impact. The future outlook involves expanding this model to other mining sites for broader applicability.

## Methodology

### Physico-mechanical properties of rock and coal

The stability of underground excavation systems depends on the behavior of the rock mass and mine openings, particularly under vibrations. Rock masses often behave like interlocking particle assemblies, exhibiting limited resistance under confinement and immersion. Mechanical properties, such as compressive strength, resist internal pressure (*p*_*0*_) applied to the rock. Tests, including point load, uniaxial, and triaxial compression, measure these properties. Analyses of coal seam No. 12 and the roof of the working space determined key physical and mechanical parameters: natural bulk density, Poisson ratio, uniaxial compressive strength, tensile strength, modulus of elasticity (*E*), soil cohesion (*C* = 1 MPa), and internal friction angle (*φ*). This reduced cohesion value reflects the expected weakening of the coal mass due to in-situ fracturing and stress redistribution in the mining environment. Analysts consistently adopted this value across both laboratory interpretation and numerical modeling to represent effective field conditions.

#### Rock sample collection and testing protocols

##### Sampling protocol

Sampling focused on lithological critical units within the roof strata of Vein No. 12 in the Datong coal mine. Cores measuring 200 × 200 × 200 mm were extracted and subsequently subdivided into smaller specimens for laboratory testing: 50 × 50 × 50 mm cubes and 50 × 50 × 100 mm rectangular prisms. These standardized dimensions ensured compatibility with uniaxial and triaxial compression testing apparatus.

##### Mechanical testing

Mechanical properties, including uniaxial compressive strength (*F*_*c*_), tensile strength (*F*_*T*_), modulus of elasticity (*E*), and Poisson ratio (*v*), were evaluated using American Society for Testing and Materials D7012 (ASTM D7012) protocols for compression tests and International Society for Rock Mechanics (ISRM) guidelines for point load testing. Triaxial compression tests yielded additional strength parameters under confined conditions. The density (*ρ*) of both coal and surrounding rock was measured, and the results were tabulated for comparative analysis (Table 1). Pore pressure and permeability (*K*) were assessed using piezometers and flow meters, with permeability calculated via Darcy’s law.

**Table 1:** Mechanical properties of coal and rock (vein no. 12).

Rock name	*ρ* (kg/m^3^)	*F*_*T*_ (MPa)	*F*_*c*_ (MPa)	*E* (GPa)	*v*
Coal	1.460	2.09	22.89	4.01	0.27
Rock	2.343	6.47	64.30	33.10	0.22

The density values listed in Table 1 are expressed in kilograms per cubic meter (kg/m^3^). The values 1.460 and 2.343 correspond to 1460 kg/m^3^ and 2343 kg/m^3^, respectively, which are typical densities for coal and rock materials.

ρ :Density of water, F_T_: tensile strength, F_c_ : compressive strength, E: modulus of elasticity, v : Poisson ratio (A.2).

#### Data analysis

Using the Hoek–Brown failure criterion, the evaluation of stress thresholds governing fracture propagation and rock failure enabled the quantification of stress redistribution patterns. Numerical simulations in Abaqus integrated these mechanical properties to model geomechanical-hydrogeological coupling, validating stress-induced permeability variations observed in laboratory testing. Key parameters, such as the elasticity modulus (*E* = 4.01 GPa for coal and 33.10 GPa for rock), were directly incorporated into simulations to ensure alignment with empirical data.

#### Integration with field observations

Stress distribution patterns near the 5302 working space revealed a measured maximum vertical stress concentration of 2.8 MPa, forming an arch-shaped profile within the goaf. This localized stress reflects post-excavation conditions, as the theoretical in-situ vertical stress at depths exceeding 800 m is typically over 20 MPa. The significant reduction is attributed to mining-induced stress redistribution, rock fracturing, and the formation of goaf areas. These observations, consistent with deep longwall mining behavior, highlight stress concentration in undisturbed pillars and stress relief in fractured zones. The findings, supported by pressure arc theory (54.5 m fracture height above the working space), were compared against steady-state load studies to emphasize the impact of dynamic vibration stress on permeability. The methodology follows ASTM/ISRM (A.2) standards, ensuring reproducibility and strong integration of empirical data with analytical modeling.

##### Operational definition of ground vibrations

Ground vibrations are subsurface oscillations caused by mechanical forces like blasting, excavation, and heavy machinery. In deep coal mines, they impact hydrogeological dynamics (A.1) by altering stress distribution, permeability, and fluid flow. Their intensity depends on geological composition, mining depth, and structural discontinuities. Mathematical modeling of these vibrations often employs formulas based on wave propagation principles, such as the basic wave equation:

1 $$\frac{{\partial^{2} u(x,z)}}{{\partial t^{2} }} = v_{speed}^{2} \nabla^{2} u(x,z)$$

where *u*(*x*,*z*) represents the displacement, *t* is time, and *v*_speed_ is the wave speed in the medium. The characteristics of ground vibrations are defined by key parameters: periodic frequency flow (*ω*), vibration amplitude ($u_{0}^{\prime }$), propagation velocity ($u_{\varepsilon }^{\prime }$), and duration. These vibrations can induce horizontal and vertical displacements (*u*_x_, *u*_z_) in the surrounding rock mass, altering subsurface stress conditions and potentially leading to instability and water loss. The relationship governing vibration displacement is given by:

2 $$u\left( {x,t} \right) = u_{0}^{\prime } cos(\omega t - n_{w} x)$$

where *n*_*w*_ is the wave number, and t represents time. Ground vibrations in mining involve stress redistribution and hydrogeological interactions. Transient vibrations from blasting generate high-stress waves, causing temporary deformation and potential rock fracturing within geological formations. Then, the displacement *u*(*x,t*) for transient vibrations follows the wave equation $u\left( {x,t} \right) = u^{\prime}_{0} e^{ - \delta t} cos(\omega t - n_{w} x)$(*δ* is attenuation). Continuous vibrations, on the other hand, result from ongoing machine operations such as drilling or conveyor belts. These vibrations exhibit lower amplitudes but prolonged exposure, leading to gradual soil compaction and settlement. The displacement function is expressed as:

3 $$u\left( {x,t} \right) = u_{0}^{\prime } cos(\omega t - n_{w} x).$$

Vibrations influence geology by causing fractures, permeability changes, settlement, and stress buildup, impacting hydrogeological systems and altering groundwater flow and stability in mining environments over time. Then, the drawdown (*Dd*) in coal seams due to vibrations is expressed as:

4 $$Dd = w\frac{\rho \phi }{{K_{c} \mu }}$$

where* w* is the well discharge, *K*_c_ is permeability, *μ* is viscosity of water, *ρ* is density of water, and *ϕ* is soil porosity (A.1). These parameters highlight the crucial role of vibrations in influencing groundwater dynamics, necessitating effective management strategies to mitigate adverse effects in deep coal mining operations.

##### Stress distribution and failure threshold in seam no. 12

Stress distribution in coal mines is influenced by fault orientation and jointed rock masses, as characterized for underground excavation design^14^. Under coal-rock combination conditions, coal fragments often flake and eject with loud sounds, resembling impact fractures beneath a hard roof^15^. Coupling mechanisms between vibrations and stress fields in mining involve wave propagation, stress redistribution, and hydrogeological responses. Dynamic stress modeling and simulations show how blasting and drilling induce deformation, creating stress concentration zones that impact permeability and groundwater flow. Hence, the dynamic stress from the vibration field (*σ*_*v*_) can be expressed as:

5 $$\sigma_{v} = G\frac{\partial u}{{\partial x}} = - G\frac{\partial u}{{\partial z}}$$

where *G* is the shear modulus. Excavation-induced stress redistribution is governed by Fourier’s law and Cauchy’s momentum equation, providing a foundation for predictive modeling of mine stability and groundwater responses. According to Cauchy’s momentum equation *σ*(*x*,*z*) + *F*(*x*) = *ρa*_c_, where *σ*(*x*,*z*) is the stress tensor, *F*(*x*) is the applied force, and *a*_*c*_ is the acceleration, stress perturbations induced by mining can generate localized concentration zones. When these stresses exceed 6 MPa, rock failure is likely to initiate. While theoretical vertical stress at depths beyond 800 m typically exceeds 20 MPa, measured stresses near the working face are often significantly lower due to excavation-induced stress relief and the presence of goaf zones. The observed vertical stress of 2.8 MPa reflects these post-failure conditions and remains consistent with established stress redistribution behavior in deep coal mining environments.

Fractures initiate and propagate based on the Hoek–Brown failure criterion can be given by $\sigma_{\max } = \sigma_{\min } + m_{i} \sqrt {\sigma_{\min } + s}$ (*σ*_max_*,σ*_min_ are principal stresses, m_i_ is a rock-dependent material constant, and s accounts for initial cohesion loss). As stresses exceed the fracture threshold, rock integrity deteriorates, expanding permeability pathways.

Based on Eq. (2), vibratory waves amplify stress redistribution, described by:

6 $$u\left( {x,t} \right) = u_{0}^{\prime } \,e^{{i\left( {n_{w} x - \omega t} \right)}}$$

These waves accumulate damage, intensifying crack expansion and destabilizing surrounding strata. Groundwater movement is governed by Darcy’s law:

7 $$q = - K_{c} \frac{dp}{{dx}}$$

The cumulative effect of fracture expansion and stress waves leads to severe groundwater depletion, impacting long-term mine stability.

**Fig. 1:**
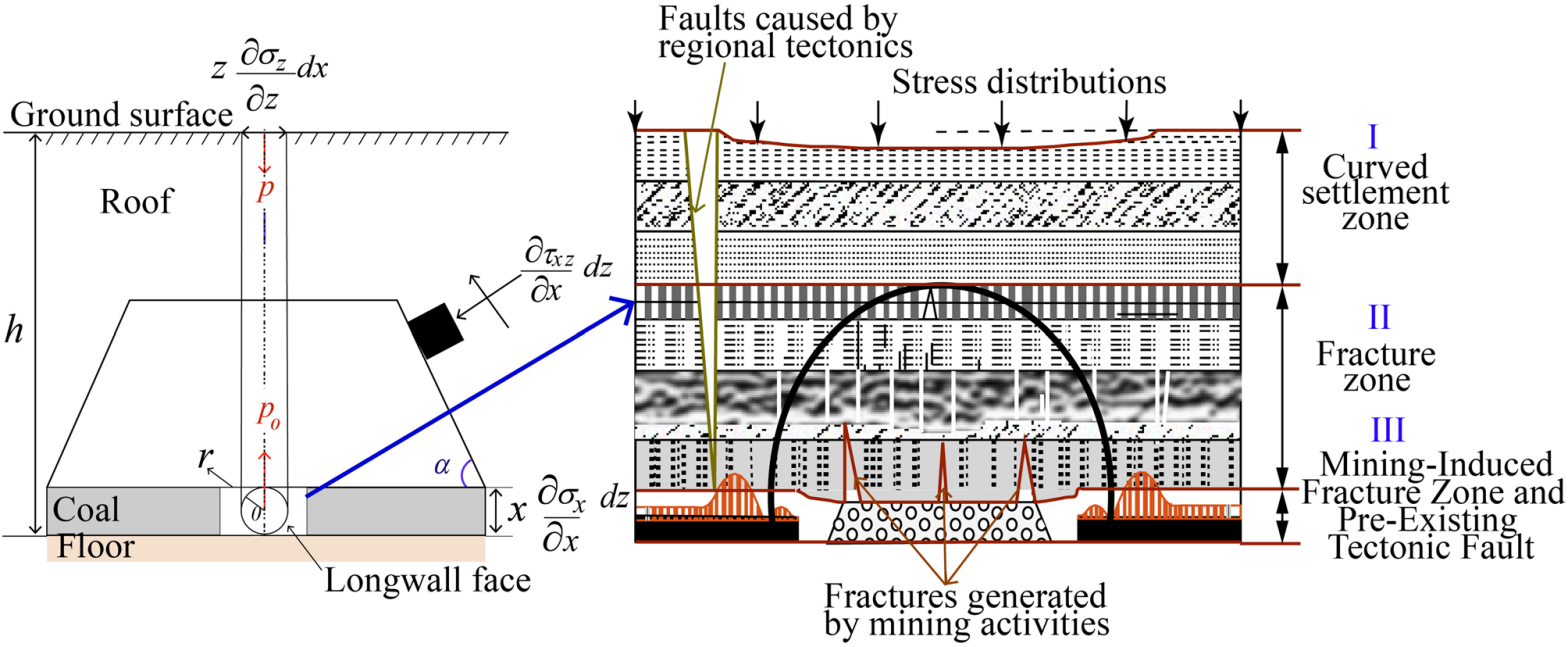
Stress distribution and fracture zones in Vein No. 12. (**a**) Geometric model of the mine, and (**b**) Pressure arch with three stress belts (I: Elastic, II: Plastic, III: Fractured)

Figure 1a depicts the geometric structure of Vein No. 12, illustrating stress distribution, fracturing, and collapse in the water-conducting zone due to mining-induced forces. Figure 1b highlights the pressure arch with three stress belts, showing how mining affects aquifer stability and groundwater flow. Using the Hoek–Brown failure criterion, it evaluates stress redistribution and vertical load impacts on rock mass stability and hydrogeological dynamics (A.1). The mechanical model illustrates the stress forces acting on the mined space under internal pressure (*p*_*0*_), external pressure (*p*), and inclination *α*. Represented on a Cartesian plane with *x* and *z* coordinates, the mine’s center experiences convergent forces between *p*_*0*_ and horizontal (*σ*_*x*_) and vertical (*σ*_*z*_) stresses. Equilibrium is expressed as $\sigma_{x} = \sigma_{z} > \sigma_{x0} = \sigma_{z0}$ and *p* > *p*_0_, highlighting the interplay of stress forces in the structural plan.

The mine roof exerts pressure and shearing forces on the longwall face, leading to a stress distribution that causes discontinuous deformation in the water-conducting zone, resulting in cracking, fracturing, and collapse. The forces generated by coal extraction are proportional to the surrounding material, which affects the aquifer and causes water to flow toward the working face through fissures, leading to water ingress incidents in the mine. The relationship among gravitational charge (*g*_*0*_), inertial force of the solid (*F*), and density of water (*ρ*) can be represented through the applied force. Additionally, the surface force density function *T*^n^(x) can be analyzed using Fourier transforms, allowing for the formulation of a linking equation between these parameters and the applied force (*F*_a_), hence we can have:

8 $$F_{a} = F + \rho .g_{0} .\;V_{s}$$

where *V*_*s*_ is the volume of the solid. This formulas indicates that the applied force is the sum of the inertial force and the buoyant force exerted by the water, which is influenced by the gravitational charge and the volume of the solid. By integrating Eq. (8) in the i–j plane, the stress components can be expressed as:

9 $$\sigma \left( {x,z} \right) = \left[ \sigma \right] = \left[ {\begin{array}{*{20}c} {\sigma_{xx} } & {\tau_{xz} } \\ {\tau_{zx} } & {\sigma_{zz} } \\ \end{array} } \right] > \left[ {\begin{array}{*{20}c} {\sigma_{{x_{0} x_{0} }} } & {\tau_{{x_{0} z_{0} }} } \\ {\tau_{{z_{0} x_{0} }} } & {\sigma_{{z_{0} z_{0} }} } \\ \end{array} } \right]$$

The interaction between the oblique traction of coal seams and faults on a triangular plane can be quantified using the force *T*^*n*^(*x*), where *e* represents strain. According to Zeng et al.^16^, the fault zone is defined by its rupture state and inclination, indicating that the rock mass behaves as a jointed rock capable of withstanding significant compression.

**Fig. 2:**
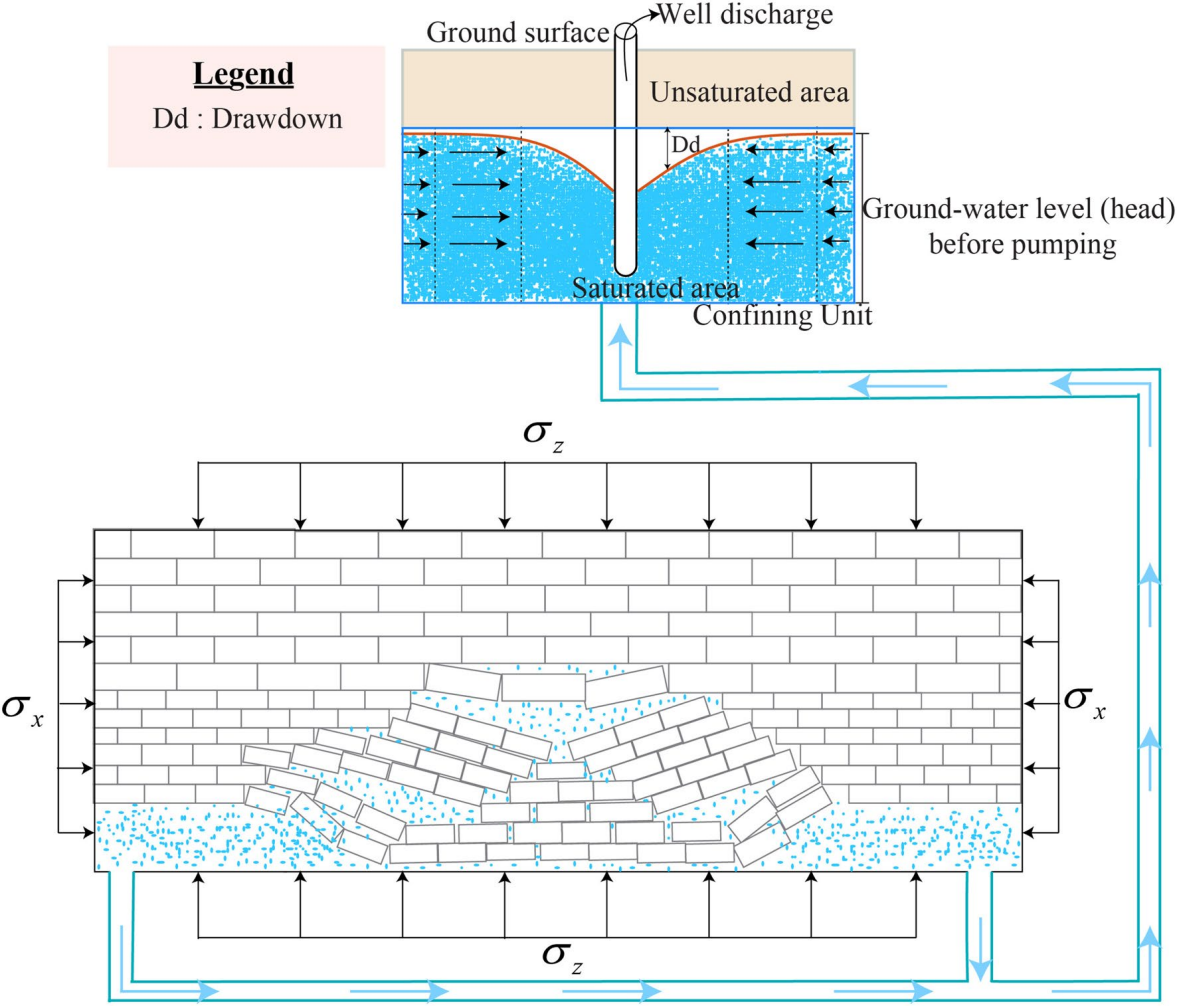
Water Flow Dynamics in Vein No. 12.

Utilizing the Hoek–Brown fracture criterion, the relationship between maximum and minimum stresses on the mine roof can be established as $\sigma \max = \sigma \min + \sqrt {\sigma \min + 1}$. When a vertical load is applied to the mine roof, the stress effects are depicted in Fig. 2. The maximum and minimum stresses can also be reformulated under normal stress conditions

10 $$\sigma_{\max } = 0.5/\left( {\sigma_{xx} + \sigma_{zz} } \right) + \sqrt {\left\{ {0.5\left( {\sigma_{xx} + \sigma_{zz} } \right)} \right\}^{2} + \left( {\tau_{xz} } \right)^{2} }$$

Given that part of the coal mine is situated beneath a sloping surface, the maximum and minimum stresses must account for a significant friction force. Consequently, the displacement vector induced at the crack layers will be evaluated based on the vertical stress, linking the mechanical behavior of the rock mass to the applied forces and stress distributions in the mining context. This analysis integrates the effects of gravitational forces, inertial forces, and fluid dynamics, providing a comprehensive understanding of the stress interactions within the mining environment.

## Hydrogeological behavior of fissured aquifers in vein no. 12

The fissured aquifer in the Datong region extends beyond the center of the rock catchment and primarily consists of coarse-grained sandstones and conglomerates. Mining activities disrupts flow paths, increasing seepage and reducing water pressure in cracks^17^. The transport fine particle follows a solid–fluid mass exchange model^18^, whereas hydraulic deterioration poses a major challenge in the maintenance of drainage systems, impacting underground structures. Darcy’s law governs the study water flow in porous coal seams, emphasizing then the importance of hydraulic gradients and conductivity. Total water flow (*w*_*T*_) can be modeled as (Fig. 2):

11 $$w_{T} = - \frac{{S_{r} \Delta p - \gamma_{\omega } }}{\mu L}K_{c} \approx K_{c} \frac{{C_{h} \mu }}{{\rho g_{0} h}}$$

where *w*_T_ in m^3^/s, $K_{c} = \rho g_{0} /\mu$ is a Permeability of the porous material (m/s), *S*_*r*_ is the cross-sectional area (*m*^2^), and Δp is the pressure gradient vector (MPa), *μ* is the viscosity of water (m^2^/s), *L* is the length of the waterfall (*m*), and *γ*_*ω*_ = *ρg*_0_ is the volumetric weight of the fluid, and *C*_h_ is the hydraulic conductivity. Total groundwater flow is influenced by key factors such as storativity (*S*), transmissivity (T) (A.1), (*T* = *C*_h_*.b* (b: thickness of the aquifer). Storativity (*S*) is defined as *S* = *V*_*T*_ /* S*_*r*_.*h*_*0*_, with *h*_*0*_ representing the variation in hydraulic head and *V*_*T*_ as the total volume of water stored), the hydraulic gradient (ΔH), water viscosity (*μ*), and flow time. However, mining-induced stress and vibrations create new fractures, increasing hydraulic conductivity in some areas while reducing permeability in compacted regions. As a result, permeability varies across mining zones, increasing in highly connected fracture networks and decreasing in compacted regions, directly affecting groundwater flow dynamics.

The average flow rate (*Q*) can be represented by *Q* = *S*_*r*_.*v*_*f*_ , where *v*_*f*_ is the flow velocity. Additionally, the total water flow (*w*_*T*_) is calculated as *w*_*T*_ = *Q.t*, where *t* is the time during which the water flow rate is measured. Hence, Fig. 2 illustrates the water flow mechanics in porous coal seams, emphasizing the interplay of hydraulic head gradients and conductivity. This highlights the aquifer’s response to mining-induced stress and changes in permeability. The negative sign indicates that the water flows from areas of high pressure to low pressure. When $p_{0} > p$, so Eq. (11) can be expressed as follows $D_{i} = - \mu \;\Delta p\;K_{c}$ (*D*_*i*_ is the discharge rate). Therefore, the flow velocity ($v_{f}$) of water in coal seams can be rewritten as:

12 $$v_{f} = - \left( {\mu \Delta p} \right)\phi \;K_{c} = \frac{q}{\phi } \approx q\left( {x,z} \right) = - \Delta p\frac{{K_{c} }}{\mu }\int\limits_{0}^{\infty } {\frac{{\partial q_{x} }}{\partial x}dz}$$

where* q* is the Darcy flux. At the boundary conditions, when the junctions of the surrounding coal and the wall mine are continuous, the density of water (*ρ*) can be defined as *ρ* = *P*/*g*_o_*h*. The elastic properties of the soil in a mine depend on its mineralogical composition. Based on the volumetric rate, permeability* K*_*c*_, hydraulic conductivity, and water storage are critical points for water protection. Then, soil porosity (*ϕ*) can be defined as follows:

13 $$\phi = \frac{{V_{v} }}{{V_{T} }} = \frac{{V_{v} }}{{V_{s} + V_{v} }} = \frac{{e_{v} }}{1 + e} = k\frac{w}{D\,d} \approx e_{v} = \frac{{V_{s} }}{{V_{v} }}$$

where *Dd* is the drawdown, *w* is the well discharge, *k* is a constant that accounts for the specific characteristics of the rock and the aquifer system, *V*_*v*_ is the volume of the air–water space, *V*_*T*_ is the total volume of water in the mine, and *e*_v_ is the void ratio. The faults could damage the integrity of the rock and influence the mechanical properties.

The faults in the overburden make the development height of the water flowing fractured zone (A.1) larger than that in normal areas, which is not good for safety underwater mining^19^. Generally, the quantity of faults per surface unit (*Q*_*fault*_), length, and throw index are needed to comprehensively describe the fault^20^. According to Fan et al.^21^, *Q*_*fault*_ = *n/surface* (number/*km*^2^).

## Theoretical analysis based on groundwater loss

### Fundamental equation

A simple method for deriving the exact solution is proposed based on the groundwater flow equation. Using the Navier-Stokes equations, the general formulas can be defined as follows:

14 $$\frac{{\partial u_{\varepsilon } }}{\partial \,t} + \left( {u_{\varepsilon } .\nabla } \right)u_{\varepsilon } = \frac{1}{\rho }\frac{{q_{{\left( {x,z} \right)}} }}{{\mu \phi^{2} K_{c} }} + \mu \nabla^{2} u_{\varepsilon }$$

According to Churchill^22^, initial flow velocity ($u_{\varepsilon }$) can be given by $u_{\varepsilon } = \left( {Dd - k\left( {w/\phi } \right)} \right)/k_{a}$. Using the Laurent series, the equilibrium equation can be established as follows:

15 $$- \frac{1}{\rho }\varphi \left( {r,t} \right) = a_{0} \rho \left( {u_{\varepsilon } .\nabla } \right)u_{\varepsilon } + \mu \nabla^{2} u_{\varepsilon } \sum\limits_{k = 0}^{\infty } {a_{K} e^{k} + \nabla p\sum\limits_{k = 0}^{\infty } {b_{K} e^{ - k} } }$$

16 $$- \frac{1}{\rho }\psi \left( {r,t} \right) = \mu \rho \nabla^{2} u_{\varepsilon } c_{0} + \Delta p\sum\limits_{k = 0}^{\infty } {c_{K} e^{k} + \left( {u_{\varepsilon } .\nabla } \right)u_{\varepsilon } \sum\limits_{k = 0}^{\infty } {d_{K} e^{ - k} } }$$

where *φ* and *Ψ* are the Goursat function, and the coefficients *a*_K_, *b*_K_, *c*_K_, and *d*_K_ are determined at the boundary condition, $\rho = E/u_{\varepsilon }^{2} r^{2}$ is the density of water (m^3^/s), and *t* is the water circulation time in the mine. With $c_{0} = 0.52\overline{a}_{0} + 0.5\mu \left( {a_{1} - b_{1} } \right)u_{\varepsilon }$, $c_{k} = - 0.26\left( {k + 1} \right)a_{k - 1} + 3.81u_{\varepsilon } \overline{b}_{k} - 0.5\mu \left( {k + 1} \right)a_{k + 1}$, and $d_{k} = 0.52\overline{a}_{k} + 0.5\mu \left( {k + 1} \right)b_{k - 1} + 1.91\left( {k + 1} \right)b_{k + 1}$.

Conformal mapping is performed on the unexcavated area. Considering a circular plane of* r*, the orthogonal projection can be established by the formulas $z = \omega \left( \rho \right) = \left( {r...r_{n} } \right)\rho$ (the derivation for can be given by $\omega^{\prime}\left( \rho \right) = \left( {r...r_{n} } \right)$. Using Eq. (9), then, stress can be given by $\omega \left( \sigma \right)/\overline{{\omega^{\prime}\left( \sigma \right)}} = \sigma$. At the boundary conditions for the surface tractions, Eq. (14) can be rewritten as:

17 $$\sum\limits_{k = - \infty }^{\infty } {A_{k} \sigma^{k} } = \mu \nabla^{2} u_{\varepsilon } \sum\limits_{k = 1}^{\infty } {a_{k} \sigma^{k} } + u_{\varepsilon } \rho \sum\limits_{k = 1}^{\infty } {\left( {k + 2} \right)\overline{a}_{k + 2} \sigma^{ - k} } - \frac{1}{\rho }\nabla p\sum\limits_{k = 0}^{\infty } {\overline{b}_{k} \sigma^{ - k} }$$

Based on the Eq. (17), the displacement boundary conditions can be obtained by:

18 $$\sum\limits_{k = - \infty }^{\infty } {C_{k} \sigma^{k} } = k\left( {u_{\varepsilon } .\nabla } \right)u_{\varepsilon } \sum\limits_{k = 2}^{\infty } {a_{k} \sigma^{k} } - \frac{1}{\rho }\nabla p\left( {ka_{1} \sigma - \overline{a}_{1} \sigma } \right) - \rho \sum\limits_{k = 0}^{\infty } {\left( {\overline{b}_{k} + \left( {k + 2} \right)\overline{a}_{k + 2} } \right)\sigma^{ - k} }$$

Hence, all constant in Eqs. (17) and (18) can be given by Table 2. The Laurent series parameters obtained allow us to propose a critical limit for stress convergence on the mine longwall face.

**Table 2:** Traction coefficients obtained under boundary conditions.

Equation (17)	Equation (18)
$a_{k} = - 0.5A_{k} ,$ k = 2, 3, 4, …,	$a_{k} = - 0.5B_{k} /k$; k = 2, 3, 4, …,
$a_{1} = - 0.26\,u_{\varepsilon } \;A_{1}$	$a_{1} = - 0.5u_{\varepsilon } \mu \left( {kB_{1} + \overline{B}_{1} } \right)/\left( {k^{2} - 1} \right)$
$b_{k} = 1.99\overline{A}_{ - k} - \left( {u_{\varepsilon } .\nabla } \right)u_{\varepsilon } \left( {k + 2} \right)a_{k + 2}$, k = 0, 1, 2, …	$b_{k} = - \mu \nabla^{2} u_{\varepsilon } \left( {\overline{B}_{ - k} + \left( {k + 2} \right)a_{k + 2} } \right)$, k = 0, 1, 2, …

### Approximate solution method based on soil dynamics

#### Justification for using the soil dynamics module

The Soil Dynamics module simulated mining-induced vibrations impacting fractured rock masses. Though designed for soil, it applies to weakened rock, like in the Datong mine, where fractures reduce stiffness. Combined with validated Drucker-Prager parameters, this approach accurately represents geomechanical and hydrogeological behaviors in mining environments. This approach strengthens the methodological transparency and scientific rigor of our study by capturing the realistic stress–strain interactions within the rock mass. The Drucker-Prager yield criterion is mathematically expressed as $f = \lambda \left( {\sigma_{x} + \sigma_{z} } \right) + 0.7\sqrt {\sigma \left( {x,z} \right) - 0.33\left( {\sigma_{x} + \sigma_{z} } \right)} - Y$ (*λ* and *Y* are material parameters dependent on cohesion (*C*) and internal friction angle *φ*, given by $\lambda = 2\sin \varphi /\sqrt 3 (3 - \sin \varphi )$ and, $Y = 3.45C\cos \varphi /(3 - \sin \varphi )$. By incorporating this pressure-dependent failure criterion, our model accurately simulates the mechanical response of fractured rock masses to mining-induced stress changes.

#### Using the Laplace transform approach

In general, unless the material is in equilibrium under the action of external pressures, it will move from the configuration specified by the displacement to a new configuration. Considering Newton’s second law, the equation of the movement of the ground mass can be expressed as $m\left( {\partial^{2} u/\partial t^{2} } \right) + k = F\left( t \right)$. Based on Eq. (13), well discharge (*w*) and drawdown (*Dd*) are influenced by flow rate, soil porosity, and displacement components. Hence, *Dd* can be given by:

19 $$Dd = \left\{ {m\left( {\partial^{2} u/\partial t^{2} } \right) - F\left( t \right)} \right\}\frac{w}{\phi } + k_{a} \left( {u_{x} + u_{z} } \right)$$

where *k*_*a*_ = *a* proportionality constant that relates drawdown to the combined effects of horizontal and vertical displacement, *F*(*t*) is the total force acting upon the mass (*N*), and *u*_x_ and *u*_z_ are the horizontal and vertical displacement, respectively. By using Eq. (19), the void ratio (*e*_*v*_) becomes:

20 $$e_{v} = \frac{1}{{k_{c} }}\left( {Dd - \left\{ {m\left( {\partial^{2} u/\partial t^{2} } \right) - F\left( t \right)} \right\}\frac{w}{\phi }\mu - k_{a} u_{\varepsilon } + k_{b} \frac{{V_{V} }}{{V_{s} + V_{T} }}} \right)$$

where *k*_*a*_, *k*_b_, and *k*_*c*_ are proportionality constants that relate drawdown to air–water space volume and void ratio, respectively. A higher flow rate increases the drawdown, while greater porosity reduces it. When *F* is composed of *P* and *P*_0_, Eq. (19) can be rewritten as $F\left( t \right) = P\left( t \right) - ku - \mu \left( {\partial u/\partial t} \right)$(* P*(t) can be found by the Ordinary Differential Equation (ODE)). By integrating an initial force of magnitude (*F*_0_) composed of a ground response time and *μ*, Eq. (19) can be rewritten as follows $u(t) = F_{0} \left( {1 - \exp ( - k(t/\mu )} \right)/k$. When $t \approx \mu /k$, the ground is returned to its original state and for *t* < *μ*/*k*, the system is extremely rigid. When *P*(t) = 0, and using Eq. (19), *F*(t) can be described by:

21 $$F\left( t \right) = - \left( {Dd - k_{a} u} \right)\frac{\phi }{w}u = \rho \frac{{\partial^{2} u}}{{\partial t^{2} }} + \mu \frac{\partial u}{{\partial t}}$$

Considering *S*_r_ subjected to a linear elastic material, the Eq. (21) can be written as $\partial \left( {\sigma S_{r} } \right)/\partial z - \rho S_{r} \left( {\partial^{2} u/\partial t^{2} } \right) = 0$(with $\sigma = E\varepsilon$ is the stress related to the Hooke’s law ($\varepsilon = \partial u\left( z \right)/\partial z$ is the strain due to vertical displacement *u*(*z*)). Then, the vertical stress can be given by $\sigma_{z} = T = E\left( {\partial u\left( z \right)/\partial z} \right)S_{r}$ ($E = \rho \left( {\partial^{2} T/\partial t^{2} } \right)/\left( {\partial^{2} u/\partial z^{2} } \right)$). Hence, Using Eq. (21), the Drucker-Prager model can be given as $f = \lambda \left( {\sigma_{x} + E\left( {\partial u\left( z \right)/\partial z} \right)S_{r} } \right) + 0.7\sqrt {\sigma \left( {x,z} \right) - 0.33\left( {\sigma_{x} + E\left( {\partial u\left( z \right)/\partial z} \right)S_{r} } \right)} - Y$.

Considering “*a*” the Laplace transforms parameter; the displacement conjugation ($\overline{u}\left( {z,a} \right)$) can be rewritten as follows:

22 $$\overline{u}\left( {z,a} \right) = \int\limits_{0}^{\infty } {u\left( {z,t} \right)} \;\exp \left( { - at} \right)dt$$

For *t* = 0, then $\partial^{2} \overline{u}\left( z \right)/\partial z^{2} = \overline{u}\left( z \right)a^{2} /u_{\varepsilon }^{2}$. In the far-field, Eq. (22) is expressed as $\overline{u}\left( z \right) = S_{r} \;\exp \left( { - az/u_{\varepsilon } } \right)$. Considering the boundary conditions *t* > *0* and *z* = *0*,* p*_*0*_ can be reformulated as $p_{0} = - E\;\partial u\left( z \right)/\partial z$. Consequently, $S_{r} = p\,u_{\varepsilon } /E\;a^{2}$, leading to $\overline{u}\left( z \right) = e^{{\left( { - a\,z/u_{\varepsilon } } \right)}} \left( {p\,u_{\varepsilon } } \right)/E\;a^{2}$. Finally, Eq. (22) simplifies to:

23 $$u\left( z \right) = u_{\varepsilon } \frac{{p\left( {t - z/u_{\varepsilon } } \right)}}{E}H\left( {t - z/u_{\varepsilon } } \right),H\left( {t - z/u_{\varepsilon } } \right) = \left\{ {\begin{array}{*{20}c} {0,\quad t < t_{0} } \\ {1,\quad t > t_{0} } \\ \end{array} } \right.t \to 0 \sim 10s$$

where H is the Heaviside unit step function.

Considering the mine depth at the free boundary stress *z* = *0*, then *z* = *h*, at *t* > *0*, the displacement (Eq. 23) at the boundary condition can be established respectively by ($\partial u\left( z \right)/\partial z = 0$ and $u\left( z \right) = u_{0}$); with $u_{0}$ being the initial displacement transform. Using the ODE, the conjugate displacement ($\overline{u}\left( z \right)$) becomes $\overline{u}\left( z \right) = \exp \left( {a\;z/u_{\varepsilon } } \right) + \;\exp \left( { - a\;z/u_{\varepsilon } } \right)$. Using the complex inversion integral and expansion of the Fourier series, $u\left( {z,t} \right)$ can be given by:

24 $$\frac{{u\left( {z,t} \right)}}{{u_{0} }} = 1 - \frac{4}{\pi }\sum\limits_{k = 0}^{\infty } {\frac{{\left( { - 1} \right)^{k} }}{{\left( {2k + 1} \right)}}} \cos \left\{ {\left( {2k + 1} \right)\frac{\pi z}{{2h}}} \right\}\cos \left\{ {\left( {2k + 1} \right)\frac{{\pi u_{\varepsilon } t}}{2h}} \right\}$$

where $\cos \left\{ {\left( {2k + 1} \right)\pi z/2h} \right\} = a/\left( {a^{2} + \left( {2k + 1} \right)^{2} } \right)$, $\cos \left\{ {\left( {2k + 1} \right)\pi u_{\varepsilon } /2h} \right\}t = a/\left( {a^{2} + \left( {\left( {2k + 1} \right)\pi u_{\varepsilon } /2h} \right)^{2} } \right)$ and $u\left( {z,t} \right)/u_{0} = 1 - 4za^{2} /\pi \left( {a^{2} + 1} \right)\left( {a^{2} + \left( {\pi u_{\varepsilon } /2h} \right)^{2} } \right)$. For *a* =—1, 0 and 1, respectively, Eq. (24) can be given as follows, $u\left( {z,t} \right) = u_{0}$, $u\left( {z,t} \right) = u_{0}$ and $u\left( {z,t} \right)/u_{0} = 1 - 4z/2\pi \left( {1 + \left( {\pi u_{\varepsilon } /2h} \right)^{2} } \right)$.

Considering a periodic frequency flow ($\omega$) of the periodic load, at *t, h*, Eq. (24) at the boundary conditions can be redefined by:

25 $$z = 0;\quad u\left( z \right) = 0$$

26 $$z = h:\;\sigma = E\frac{\partial u\left( z \right)}{{\partial z}} = p_{0} \sin \left( {\omega \;t} \right)$$

where $\sin \left( {\omega \;t} \right) = \omega /\left( {a^{2} + \omega^{2} } \right)$. Then $u\left( z \right) = F\left( z \right)\sin \left( {\omega \;t} \right)$. Using Eq. (21), the condition establishes by the function *F*(z) can be $\partial^{2} F/\partial z^{2} = - \omega^{2} F/u_{\varepsilon }^{2}$. The Final validation of Drucker-Prager model is $f = \lambda \left( {\sigma_{x} + p_{0} \sin \left( {\omega \;t} \right)S_{r} } \right) + 0.7\sqrt {p_{0} \sin \left( {\omega \;t} \right) - 0.33\left( {\sigma_{x} + p_{0} \sin \left( {\omega \;t} \right)S_{r} } \right)} - G$ (wih *z* = *h*). And then,

27 $$u\left( {z,t} \right) = - \frac{{p_{0} u_{\varepsilon } }}{E\omega }\frac{{\sin \left( {\omega \;z/u_{\varepsilon } } \right)}}{{\cos \left( {\omega \;h/u_{\varepsilon } } \right)}}\sin \left( {\omega \;t} \right)$$

Using *u*(z) = *u*_0_, the result can be given as $u\left( {h,t} \right) = \int {u_{0} \sin \left( {\omega \;t} \right)} \,dt$ with $u_{0} = - p_{0} u_{\varepsilon } /E\omega \tan \left( {\omega \;h/u_{\varepsilon } } \right)$. Hence, the initial force of magnitude (*F*_*0*_) can be rewritten as:

28 $$F_{0} = \omega u_{\varepsilon } \frac{{ES_{r} }}{{u_{\varepsilon } }}cotu_{\varepsilon } \cdot cot\omega h$$

where $$\omega = \omega_{k} = \left( {2k + 1} \right)\pi u_{\varepsilon } /2h$$,$$\tan \left( {\omega \;h/u_{\varepsilon } } \right) = \left( {\omega \;h/u_{\varepsilon } } \right)\left\{ {a^{2} + \left( {\omega \;h/u_{\varepsilon } } \right)^{2} } \right\}/a\left\{ {a^{2} + \left( {\omega \;h/u_{\varepsilon } } \right)^{2} } \right\}$$ and $$k = 0,1,2,...$$.

#### Displacement evaluation based on overlapping coal seams

Considering that vibrations caused by excavation machinery and other internal variations have a frequency and the overlap period (*ζ*), the displacement on the horizontal plane can be introduced by $u\left( {x,t} \right) = u^{\prime}_{0} \cos \left\{ {\omega \,\left( {t - x/u^{\prime}_{\varepsilon } } \right)} \right\}$($u^{\prime}_{0} = - p_{0} u^{\prime}_{\varepsilon } /E\tan \left( {\omega \;h/u^{\prime}_{\varepsilon } } \right)\omega$, $u^{\prime}_{\varepsilon } = \left( {G/\rho } \right)^{0.5} /r^{2}$ is the propagation velocity, *G* is the shear modulus and *ω* = *2πζ*. Using Eq. (19), the drawdown (*Dd*) in the coal seams can be expressed as:

29 $$Dd = k\frac{w}{\rho \phi }\mu$$

Using Eq. (11) through Darcy’s Law, Total water flow (*w*_T_) can be rewritten as:

30 $$w_{T} = S.T.\frac{{\Delta h_{1} - \Delta h_{2} }}{\mu }t$$

**Fig. 3:**
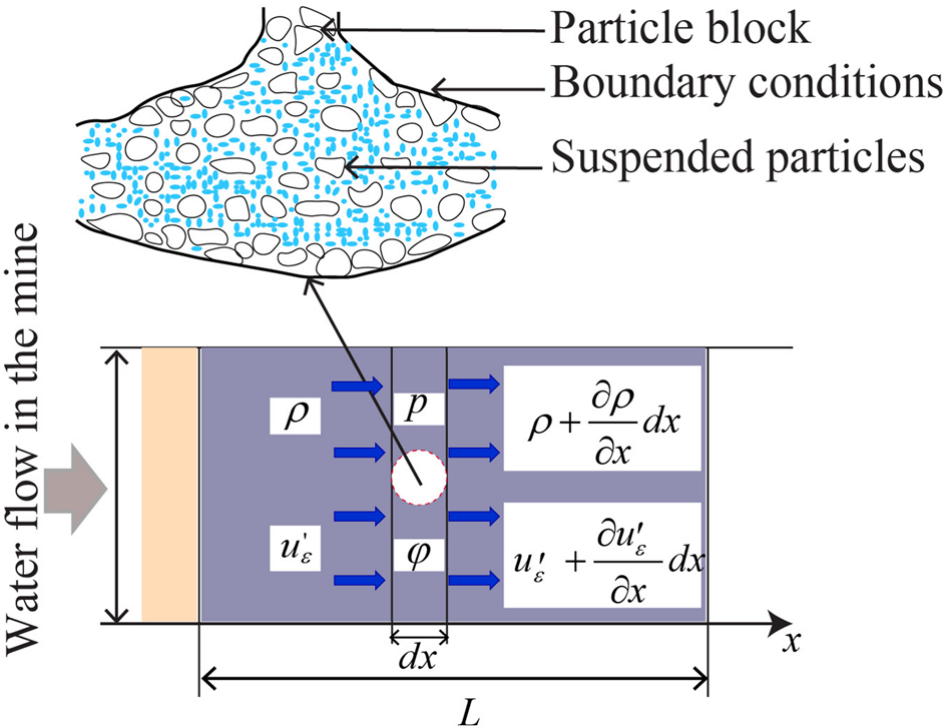
Diagram of water filtration in a porous medium (A.2).

With $\mu = Kh\left( {\Delta h_{1} - \Delta h_{2} } \right)/v_{f} L$. Then, Eq. (30) becomes $w_{T} = S.T\left( {v_{f} L/Kh} \right)t$. Hence, the displacement component can be written as:

31 $$u\left( {x,t} \right) = \left\{ {m\left( {\partial^{2} ux/\partial t^{2} } \right) - F\left( t \right)} \right\}\frac{{u_{0}^{\prime } u_{\varepsilon }^{\prime } \omega_{v} }}{G\rho }\zeta \cdot \sin \left( {\omega ,t} \right) + w_{T}$$

where *ω*_v_ is the frequency of the vibration. During unloading for homogeneous layers, the following conditions *z* = *h*; $u\left( {x,t} \right) = u_{0}^{\prime } \sin \left\{ {\omega \,\left( {t - x/u_{\varepsilon }^{\prime } } \right)} \right\}$ and $z = 0$, $\partial u\left( z \right)/\partial z = 0$ can be considered as the boundary condition. Hence, $\partial u_{z} /\partial x < \partial u_{x} /\partial z$. Based on the mass of the surface load as the equivalent thickness of the soil layer, the shear stress (*τ*(*x*,*z*)) is given by $\tau \left( {x,z,t} \right) = \rho^{2} \partial^{2} u\left( {x,t} \right)/\partial t^{2} = G\partial u\left( {x,z} \right)/\partial z$ (Fig. 3). Structural vibration-induced ground shaking is mainly subject to kinematic stresses due to interaction with the deforming soil^23^ (Eq. 31). Then, using Verruijt^24^, Eq. (31) becomes:

32 $$\frac{{u\left( {x,t} \right)}}{{u_{0}^{\prime } }} = \frac{z}{h}\frac{{\left\{ {a - \rho \left( {\omega /u_{\varepsilon } } \right)^{2} } \right\}}}{{\left\{ {a^{2} + \left( {\omega z/u_{\varepsilon } } \right)^{2} } \right\}}}\frac{{\left\{ {a^{2} + \left( {\omega h/u_{\varepsilon } } \right)^{2} } \right\}}}{{\left( {a - h\left( {\omega /u_{\varepsilon } } \right)^{2} \rho } \right)}}\left\{ {\left( {\frac{\omega }{{a^{2} + \omega^{2} }}} \right) - x\frac{{\omega x/u_{\varepsilon } }}{{a^{2} + \omega x/u_{\varepsilon }^{\prime 2} }}} \right\}$$

Displacement around excavations depends on mass, diameter, and flow frequency, with infiltration and permeability governed by Darcy’s law^24^. For the small periodic frequency flow $\omega = 1.6\,u_{\varepsilon } /h \approx 2\pi /\zeta$,$\zeta = 4h/u_{\varepsilon }$ and $G = 0.5\lambda \sigma_{z}$, with *λ* is the compression coefficient of the soil. Then, *u*(*z*) can be given by:

33 $$u\left( {z,t} \right) = \frac{{u_{0}^{\prime } \left( {\Delta h_{1} - \Delta h_{2} } \right)t}}{{\cos \left( {\omega \,h/u_{\varepsilon } } \right) - \left( {\omega \rho /u_{\varepsilon } \,} \right)\sin \left( {\omega \,h/u_{\varepsilon } } \right)t}} + \frac{S}{\mu }T$$

Using $q_{x} = - \left( {\partial u_{x} /\partial x} \right)K_{c} /\mu$, $q_{z} = - \left( {\partial u_{z} /\partial z} \right)K_{c} /\mu$ and $q_{xz} = - K_{c} /\mu \left( {\partial u_{z} /\partial x + \partial u_{x} /\partial z} \right)$, the Darcy flux can be given by:

34 $$q\left( x \right) = u_{0}^{\prime } \left( {K_{c} /\mu \;u_{\varepsilon }^{\prime } } \right)\omega \cos \left( { - \frac{x\omega }{{u_{\varepsilon }^{\prime } }}} \right)\frac{{\cos \left( {\omega \,z/u_{\varepsilon } } \right) - \left( {\omega .m/u_{\varepsilon } } \right)\sin \left( {\omega \,z/u_{\varepsilon } } \right)}}{{\cos \left( {\omega \,h/u_{\varepsilon } } \right) - \left( {\omega \,m/u_{\varepsilon } } \right)\sin \left( {\omega \,h/u_{\varepsilon } } \right)}}$$

35 $$q\left( z \right) = - \frac{{2u_{0} \pi K_{c} }}{\mu }\frac{{a\left( {\left\{ {m\left( {\partial^{2} u/\partial t^{2} } \right) - F\left( t \right)} \right\} + 0.5} \right)z\left( { - 1} \right)^{{\left\{ {m\left( {\partial^{2} u/\partial t^{2} } \right) - F\left( t \right)} \right\}}} }}{{\left( {a^{2} h^{2} + \left( {\left( {\left\{ {m\left( {\partial^{2} u/\partial t^{2} } \right) - F\left( t \right)} \right\} + 0.5} \right)\pi u_{\varepsilon } } \right)^{2} } \right)\left( {a^{2} h^{2} + \left( {\pi \left( {\left\{ {m\left( {\partial^{2} u/\partial t^{2} } \right) - F\left( t \right)} \right\} + 0.5} \right)z} \right)^{2} } \right)}}$$

For *z* = *0*, Eq. (35) can be rewritten as $q_{xz} = \Delta dK_{c} \left( {A_{s} \Delta p - \gamma_{\omega } } \right)L\,\sin \omega \left( {t - x/u_{\varepsilon }^{\prime } } \right)$. Referring to mathematical evaluation (for $\omega \approx 0$), the displacement becomes,$u = u^{\prime}_{0}$ with $a \approx 0.1$. Depending on the fracturing plane of the coal body, the Darcy parameters (Eqs. 34, 35) are obtained based on the rock displacement, allowing the conclusion that the flow directions of water in the mine can be related to the degree of fracturing of the rock and the depth of the mine. Having established the geological context, we now detail the experimental protocols.

## Modeling and results

### Geological and structural features of the Datong basin

#### Geological context of the Datong basin

The Datong Coal Basin, located in southern Yinshan, spans an area of 1,900 km^2^ and is framed by the Pingwang-Emaokou fissures, the Mount Lvliang syncline, and the Mount Hongtao syncline. The basin’s geological development, driven by Caledonian tectonic activity in the early Paleozoic, resulted in the formation of stable Carboniferous, Permian, and Jurassic coal beds. Cretaceous deposits subsequently covered these strata during the Yanshan period, consolidating them and forming pseudo-consolidated layers characterized by minimal tectonic disruptions. The stable fault layouts observed throughout the basin have largely limited significant tectonic activity^25–28^. This stability has contributed to the region’s geological maturity and stratigraphic features, which underpin the area’s hydrological behavior and groundwater dynamics.

#### Structural features of vein No. 12 and hydrological implications

**Fig. 4:**
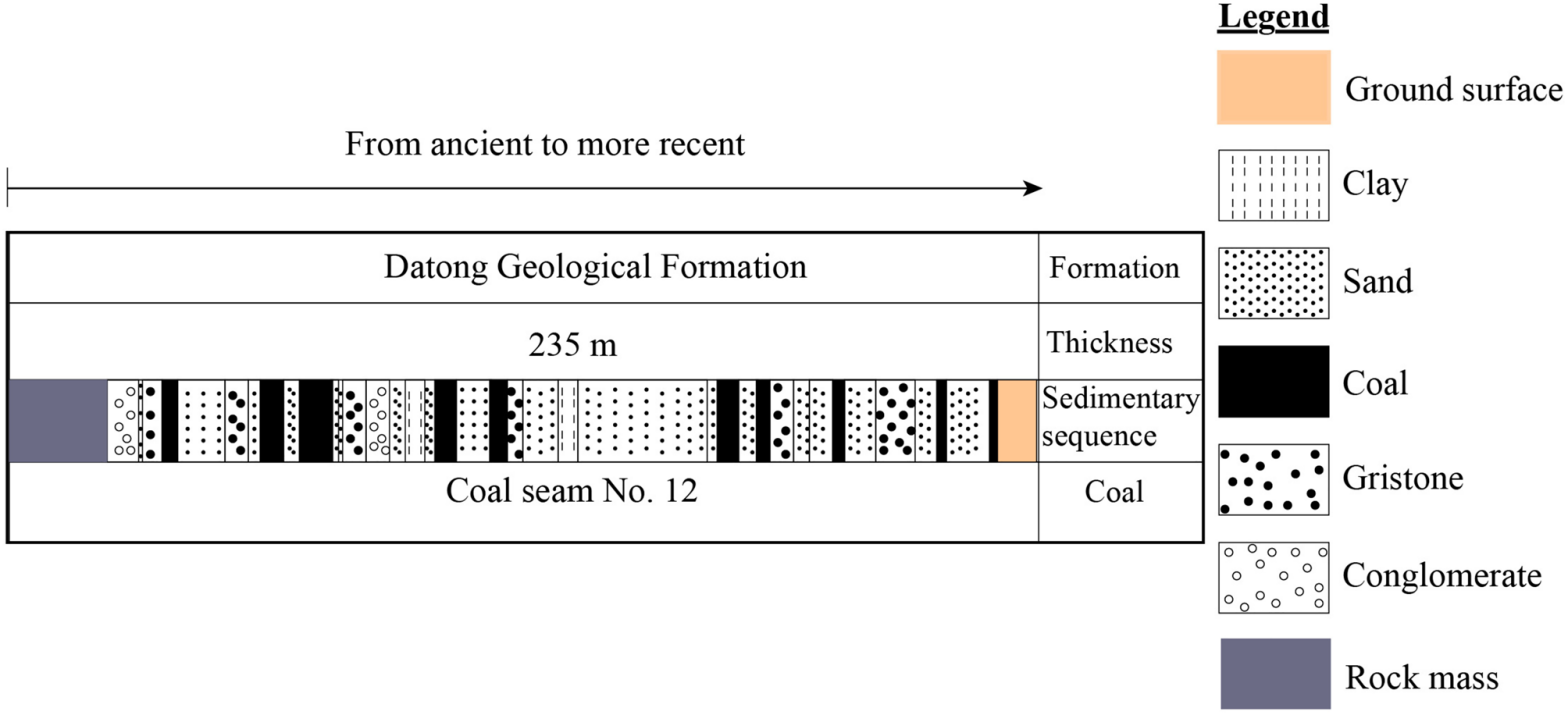
Stratification of Vein No. 12.

**Fig. 5:**
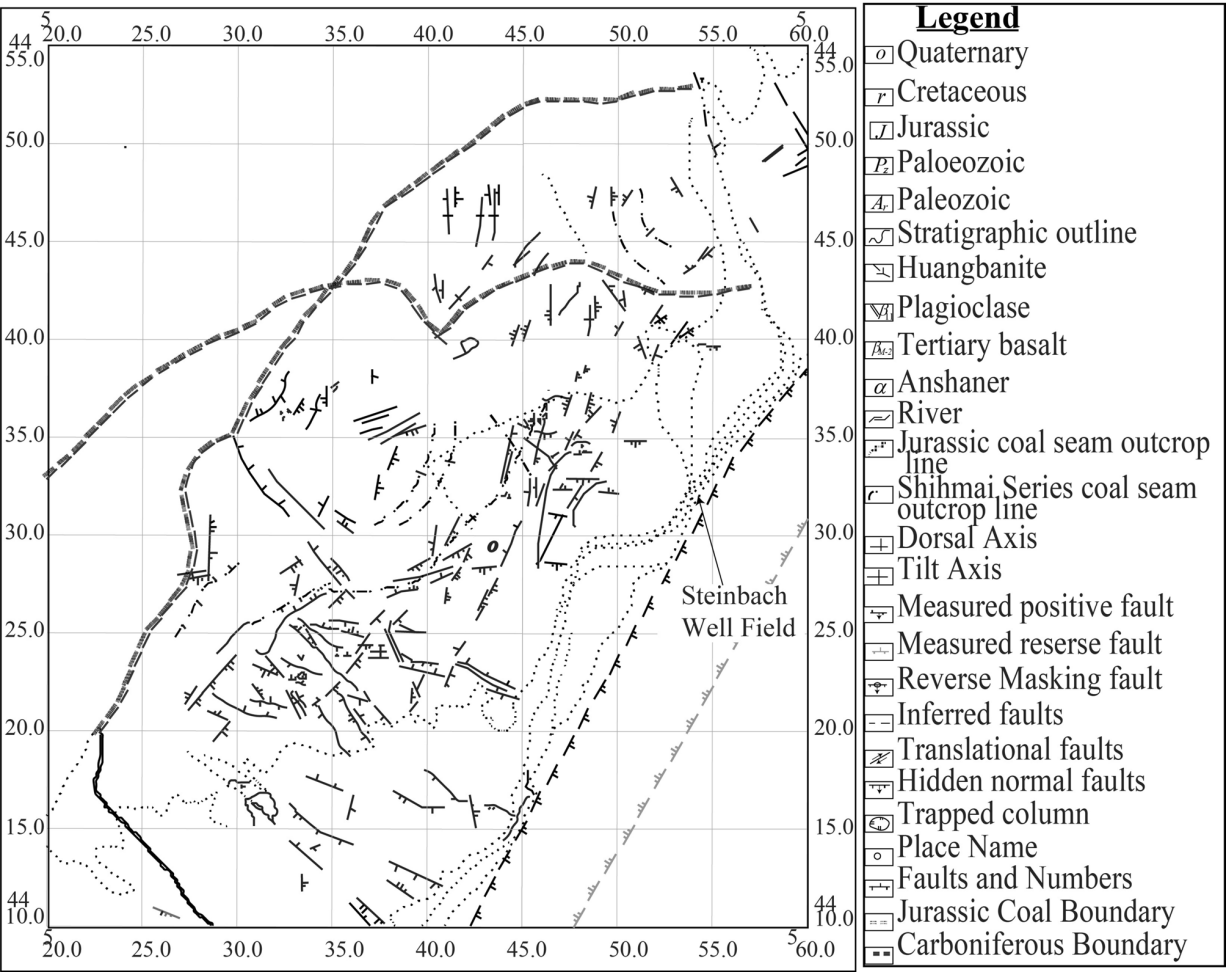
Geological Structure of the Datong Mine.

Vein No. 12 of the Datong Coal Basin exhibits a monoclinic structure, with strata inclined at N10-50°E and dips ranging from 3 to 10*°* in the central area, increasing to steeper angles of 30–80*°* in the southeast. Table 3 highlights fault characteristics, including orientation, dip angles, and altitude differences, which facilitate the propagation fissure water into voids, thereby reducing water retention within aquifers. Figure 4 illustrates the stratification of Vein No. 12, while Fig. 5 showcases the lithological formations of the region, emphasizing the interplay between brittle coal (Mohs hardness 2–3) and sandstone layers. These materials fracture under tectonic stress, creating pathways for water loss. Fissures and fractures not only redirect groundwater but also significantly decrease aquifer stability and water flow, underscoring their critical role in the hydrological dynamics of the mine. Effective management strategies must consider these structural and geological nuances to mitigate water depletion and ensure sustainable resource utilization.

**Table 3:** Fault statistics of mine field area.

Fault location	Fault orientation			Difference in altitude (m)	Nature
	Strike	Dip	$\alpha$(°)		
Xipan District return air tunnel	NNE	NWW	54	0–4	Normal fault
Southern part of the DF27 fault	SWW	NNW	60–63	9–67	Normal fault
Northwestern part of the well field	SWW	SSE	60–68	0–69	Normal fault
Southern part of the DF29 fault	NWW	NNE	59	12–15	Normal fault

α is the inclination (°).

### Hydrogeological dynamics of the Datong coal mine

#### Geological overview and impact of fractures on groundwater movement

The Datong coal mine is situated within a complex geological framework characterized by nearly horizontal coal seams, with a total thickness of 235 m. Coal seam No. 12, part of the Datong formation, varies in thickness from 0.35 to 7.90 m, averaging 2.80 m^26,29^. The area’s lithology primarily consists of clay, sand, conglomerate, sandstone, and coal (Fig. 5). Fractures and fissures in the mine play a crucial role in governing groundwater movement, impacting water flow based on their orientation, permeability, and distribution. These geological features can either enhance or obstruct groundwater migration, as fractures create preferential flow paths that alter the hydraulic conductivity of the strata^30,31^. Additionally, fractures can lead to erosion, particularly in saturated silty sands, influencing local hydrological dynamics^32^. The coal seams are embedded within a northwest-dipping monoclinic structure, where surface faults such as those in the Xipan district and the southern parts of DF27 and DF29 affect ground vibrations and water flow. Table 3 provides detailed geological parameters influencing the basin’s structural and hydrological configuration. While these parameters represent intact material conditions, the numerical simulation applied slightly different values to better reflect in-situ coal behavior under mining-induced stress changes. Specifically, coal parameters such as a tensile strength of 0.3 MPa and a Poisson ratio of 0.35 were adopted. These values differ from the experimentally measured tensile strength of 2.09 MPa and Poisson ratio of 0.27 presented in section "Methodology". The discrepancy is intentional: while laboratory tests characterize intact coal, the simulation intentionally represents the post-mining, fractured condition of the coal mass, where dynamic loading and excavation-induced stress redistribution degrade material properties. The use of reduced values reflects field-observed weakening in deep mining environments, where microcracking, vibration damage, and stress concentration significantly reduce coal’s effective mechanical performance. This adjustment ensures that the numerical model accurately simulates the behavior of damaged coal under realistic geomechanical conditions.

#### Hydraulic effects of fractures and fissures in the datong mine

##### Influence of fractures on groundwater movement

Fractures in the Datong Mine, both natural and mining-induced, play a dual role in groundwater dynamics. While they create preferential flow paths that facilitate water movement, excessive fracturing can lead to permeability reduction due to compaction and clogging by fine particles^31^. Recent studies indicate that fractures, combined with the low permeability of coal seams, significantly hinder vertical water migration. Geological surveys and geophysical mapping show that permeability can decrease by up to 60%^30^, affecting water storage and movement.

This permeability loss varies with fracture orientation, connectivity, and aperture size. In some zones, fractures act as barriers, preventing water from entering mining areas and complicating dewatering, particularly where sandstone fissure aquifers have low water content (Tables 4, 5). As a result of permeability loss, water movement within the mine becomes highly unpredictable, requiring advanced dewatering strategies to prevent excessive accumulation or depletion.

**Table 4:** Hydraulic impact of factures in Datong mine.

Fracture type	Orientation	Permeability (%)	Impact on groundwater
Natural fracture	NNE-SSW	−20	Moderate water increase
Induced fracture	Varies	−60	Significant permeability reduction
Large faults	NW–SE	10	Creates water pathways

**Table 5:** Roof and floor conditions of working face.

Rock name		H (m)	Thickness (m)	Lithological characteristics
Clay	Basic top	92.30	5.66	Thick layered structure composed of feldspar and quartz
Sand		98.53	6.23	Off-white coarse particles, mainly quartz and feldspar
Mudstone		99.99	1.46	Dark grey, muddy structure, thin plate-like structure
Fine-grained sandstone	Direct top	103.77	3.78	Light grey, massive, mainly composed of quartz, semi-hard
Coal		113.00	7.5–9.1	Locally developed fissures, the surface coal seam are mainly long-flame coal and semi-coking coal
Mudstone	Direct bottom	114.04	1.04	Grey massive, uniform in texture, brittle, flat fracture

*h* is depth.

The internal friction angle (*φ*) and bulk modulus of coal and surrounding materials (Table 6) are critical for stability analysis. Although the cohesion and elastic modulus used in the simulation (*C* = 1.0 MPa, *E* = 12.5 GPa) differ from the laboratory-measured values (*C* = 2.5 MPa, *E* = 4.01 GPa for coal and 33.10 GPa for rock), they were deliberately adjusted to represent the fractured and vibration-degraded condition of the in-situ coal-rock mass. These effective parameters account for stress redistribution, heterogeneity, and mechanical weakening not captured in intact sample testing. The model was calibrated to reproduce observed field responses such as stress evolution, permeability variation, and displacement ensuring consistency between simulation outputs and measured behavior.

**Table 6:** Sedimentary sequence and material properties of coal seam no. 12.

Sedimentary sequence	Thickness (m)	Internal friction angle (°)	Bulk modulus (MPa)	Effective elastic modulus (GPa)
Clay, Sand, Conglomerate, Gristone, and Coal	235	20	1.3	12.5

The Effective Elastic Modulus represents the value of the mass of coal and fractured rock used in the simulations. Laboratory measurements of intact coal and rock yielded values of 4.01 GPa and 33.10 GPa, respectively.

Stress analysis reveals vertical stress values of 6 MPa at* t* = 10 s and 2.10 MPa at *t* = 5 s (Table 7), aiding hydromechanical assessments. Fractures significantly reduce permeability by up to 50%, especially in active mining zones, leading to water stagnation and impacting groundwater movement and coal seam stability. However, fractures also facilitate water movement within voids created by mining activities, influencing the hydraulic balance in the mine. Understanding these dynamics is critical for effective water control strategies in deep coal mining environments.

**Table 7:** Stress variations induced by mine roof movement.

Stresses	For time $t \to 0 - 10$										
	0	1	2	3	4	5	6	7	8	9	10
*σ*_x_	0	0.10	0.20	0.39	0.68	1.05	1.32	1.75	2.16	2.52	3.00
*σ*_z_	0	0.20	0.40	0.78	1.36	2.10	2.64	3.50	4.32	5.04	6.00
*τ* (x,z)	0	0.01	0.10	0.20	0.34	0.53	0.66	0.88	1.08	1.26	1.50

σ_x_ is the Horizontal stress (MPa),σ_z_ is the Vertical stress(MPa), and τ(x,z) is the Shear stress (MPa).

Although the bulk modulus listed here (1.3 MPa) is significantly lower than the theoretical estimate of approximately 2.9 GPa derived from measured material parameters, the simulation deliberately uses this value to represent an effective bulk modulus of the fractured rock mass. This lower value reflects the reduced stiffness due to extensive fracturing, porosity increase, and stress softening under dynamic mining conditions. It is calibrated to better capture the hydro-mechanical behavior observed in the field and laboratory, rather than modeling the intact rock alone.

#### Ground vibration and their effect on groundwater flow

Mining activities, particularly blasting and excavation generate ground vibrations that significantly influence fracture stability and groundwater flow. These vibrations alter stress distributions within rock formations, which can either increase permeability by opening fractures or decrease it by causing compaction. The vertical displacement of coal during extraction further amplifies these effects.

Groundwater loss in mines is intricately linked to vertical movements, with water seeping into newly formed voids and settlement zones. Excavation-induced stress reduces internal pressure, influencing surface dynamics, with stress values ranging from 0.40 to 926.73 MPa over a flow time of 0 to 10 s. The average horizontal stress is 1.29 MPa, vertical stress is 2.40 MPa, and shear stress is 0.595 MPa, as detailed in Table 7.

**Fig. 6:**
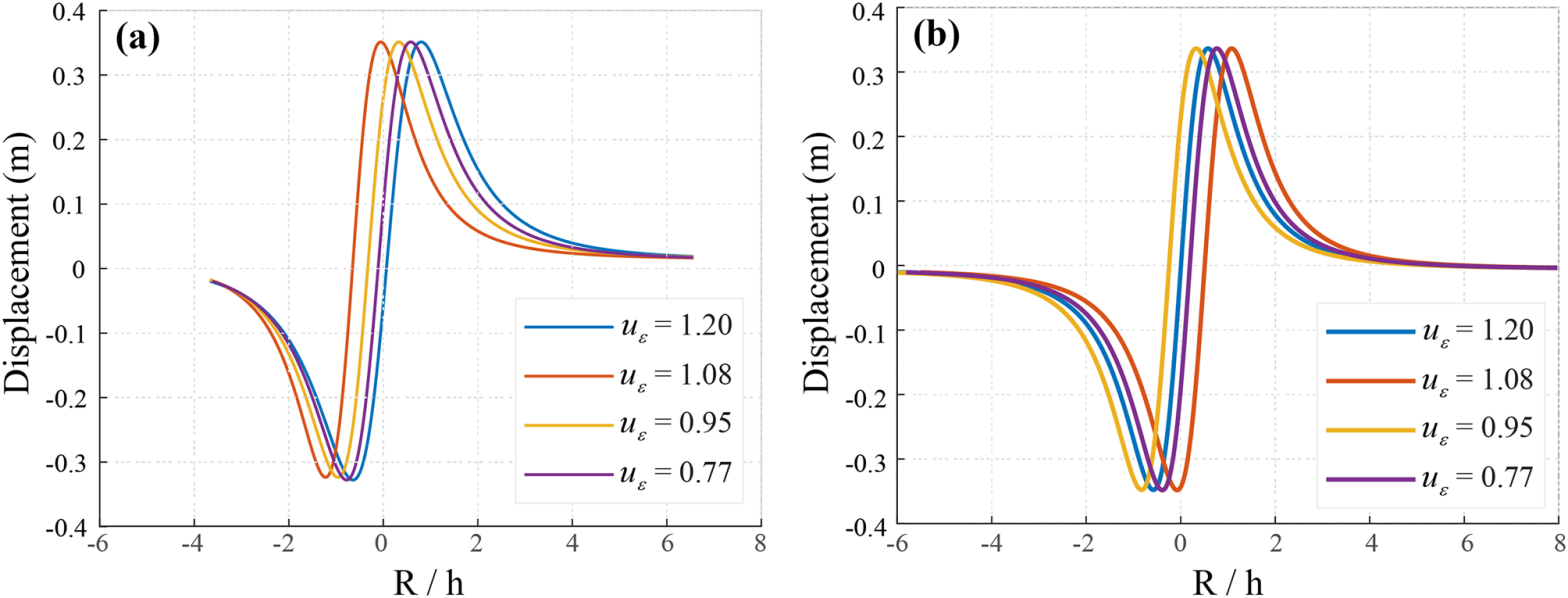
Ground Vibrations and Their Impact on Groundwater Dynamics (R/h is the Ratio of radial distance to depth). (**a**) – 3.5 < R/h < 6.8 and (**b**) – 5.9 < R/h < 8.

**Fig. 7:**
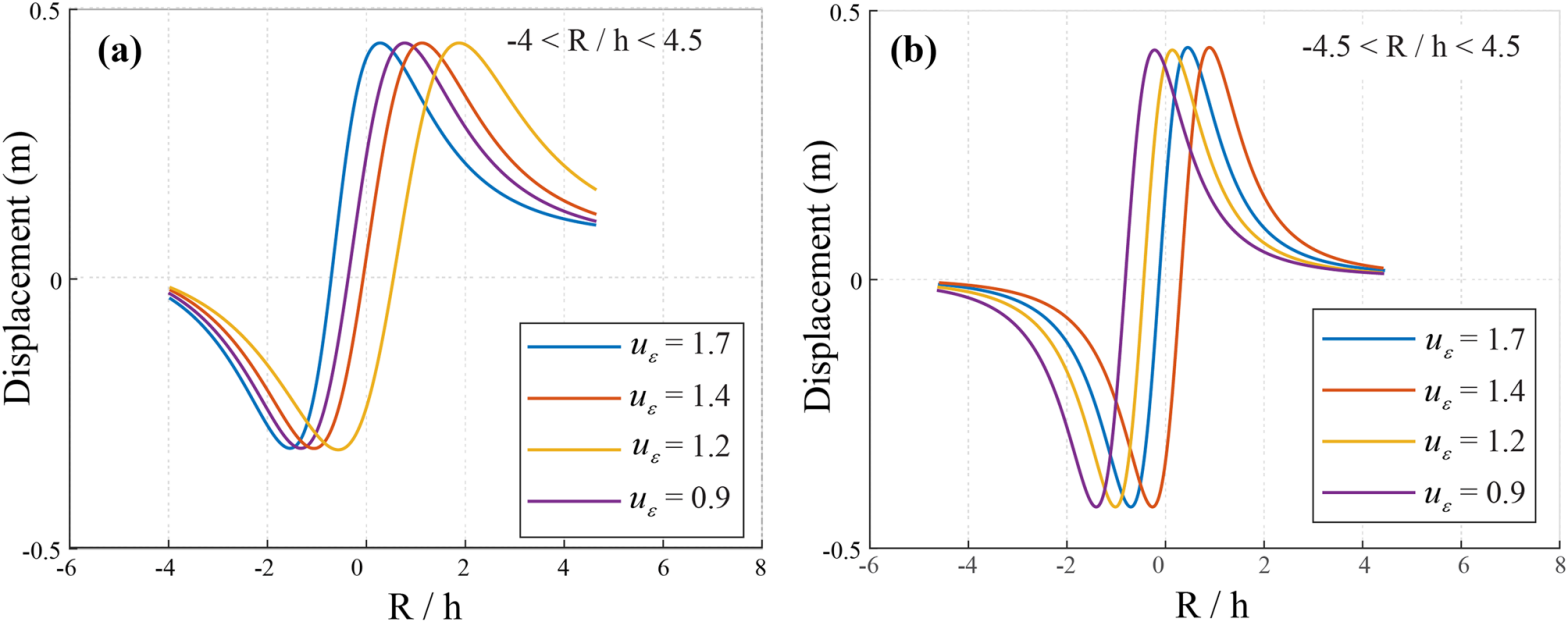
Vertical Displacement due to Mining Vibrations (R/h is the Ratio of radial distance to depth). (**a**) − 4 < R/h < 4.5, and (**b**) − 4.5 < R/h < 4.5.

Vibrations and stress redistribution significantly impact groundwater pathways by widening fractures and modifying permeability. Over time (*t* = 0 to 10 s), stress levels increase due to vibratory fluctuations. In compacted zones, these vibrations can lower permeability, restricting groundwater movement, while in some cases; they may enhance permeability, allowing water infiltration into mining voids. The effects of vibration and vertical displacement lead to inconsistent water retention and loss patterns (Fig. 6), with maximum displacements ranging from −0.35 m to 0.35 m and −0.44 m to 0.41 m, as shown in Fig. 7.

**Fig. 8:**
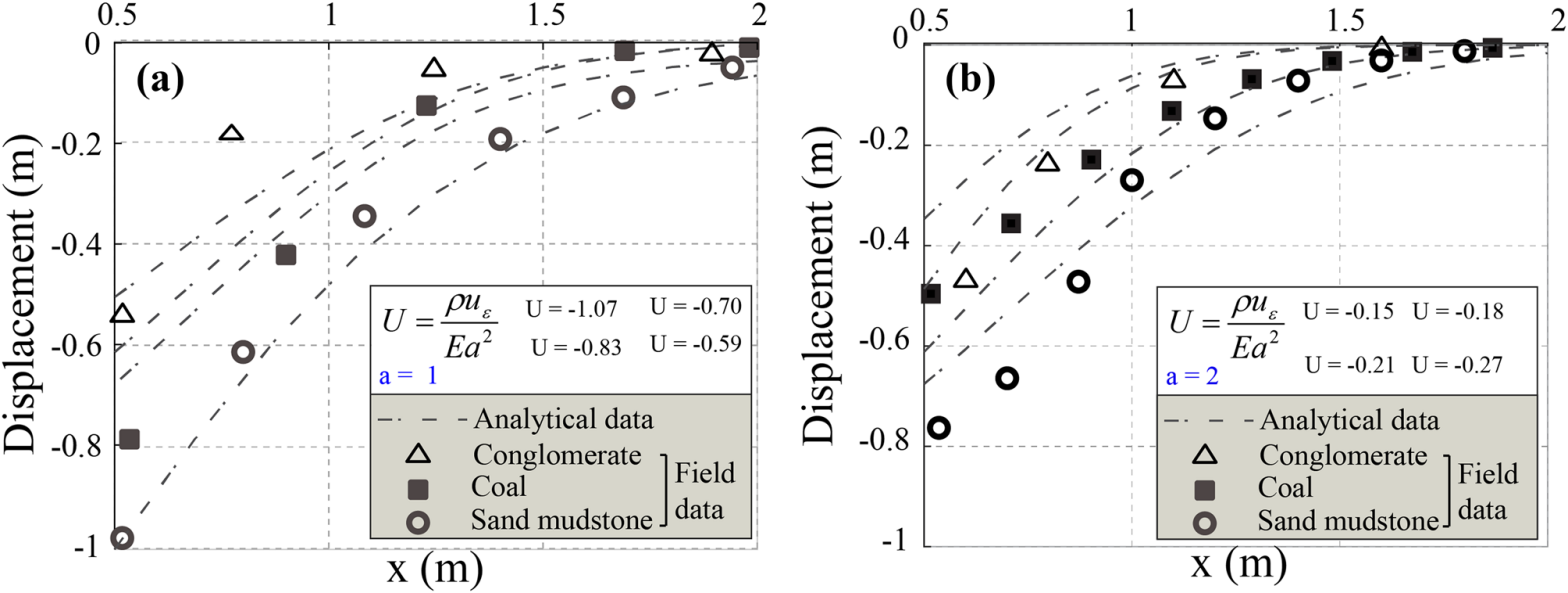
Ground Settlement Induced by Stress Redistribution (*a* is the Laplace transform coefficient). (**a**) *a* = 1, and (**b**) *a* = 2.

Ground settlement, induced by stress redistribution during mining operations (Fig. 8), typically occurs when geological structures experience stress levels exceeding 6 MPa. As coal extraction progresses, these stress changes lead to increased settlement, which may compromise structural stability and alter groundwater flow dynamics. Effective monitoring and management strategies are essential to mitigate these adverse effects. Understanding these interactions is crucial for designing robust groundwater management systems in deep coal mines.

**Fig. 9:**
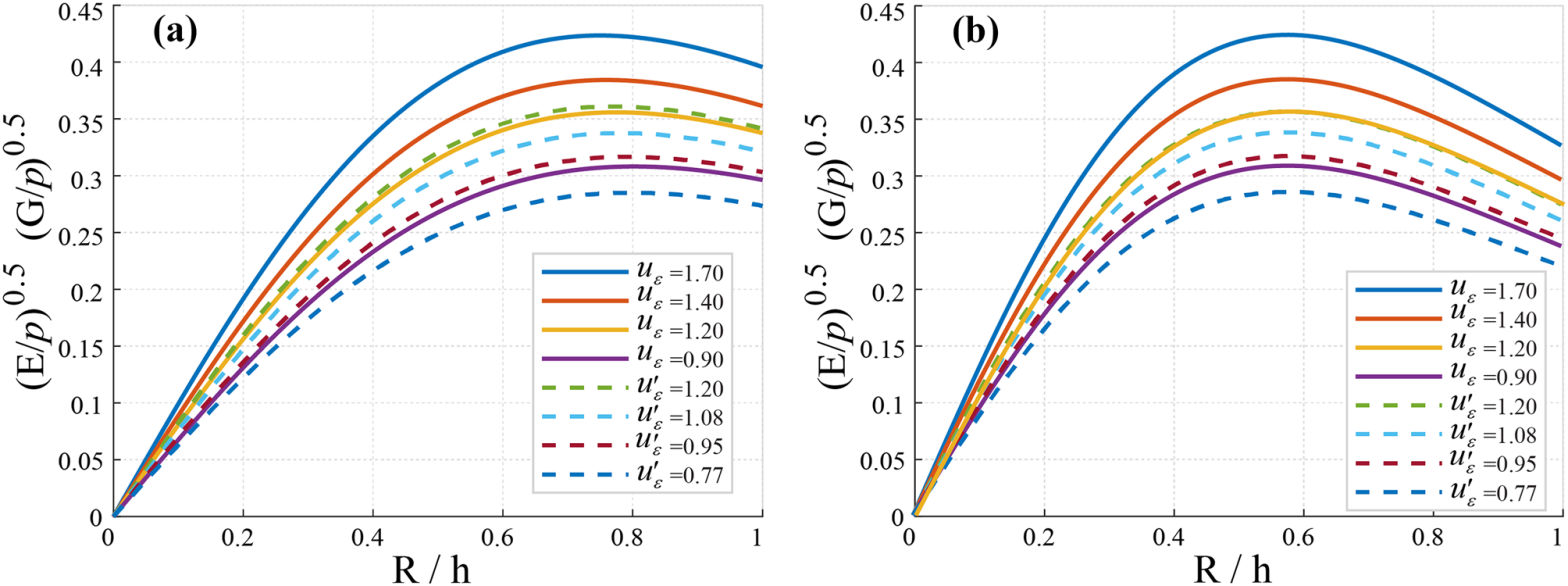
Relationship between the initial flow velocity (*u*_ε_) and propagation velocity ($u^{\prime}_{\varepsilon }$) (A.2). (**a**) R/h = [0, 1], *u*_ε_ = 0.35 and *u*'_ε_ = 0.31, and (**b**) R/h = [0, 1], *u*_ε_ = 0.29 and *u*'_ε_ = 0.23.

**Fig. 10:**
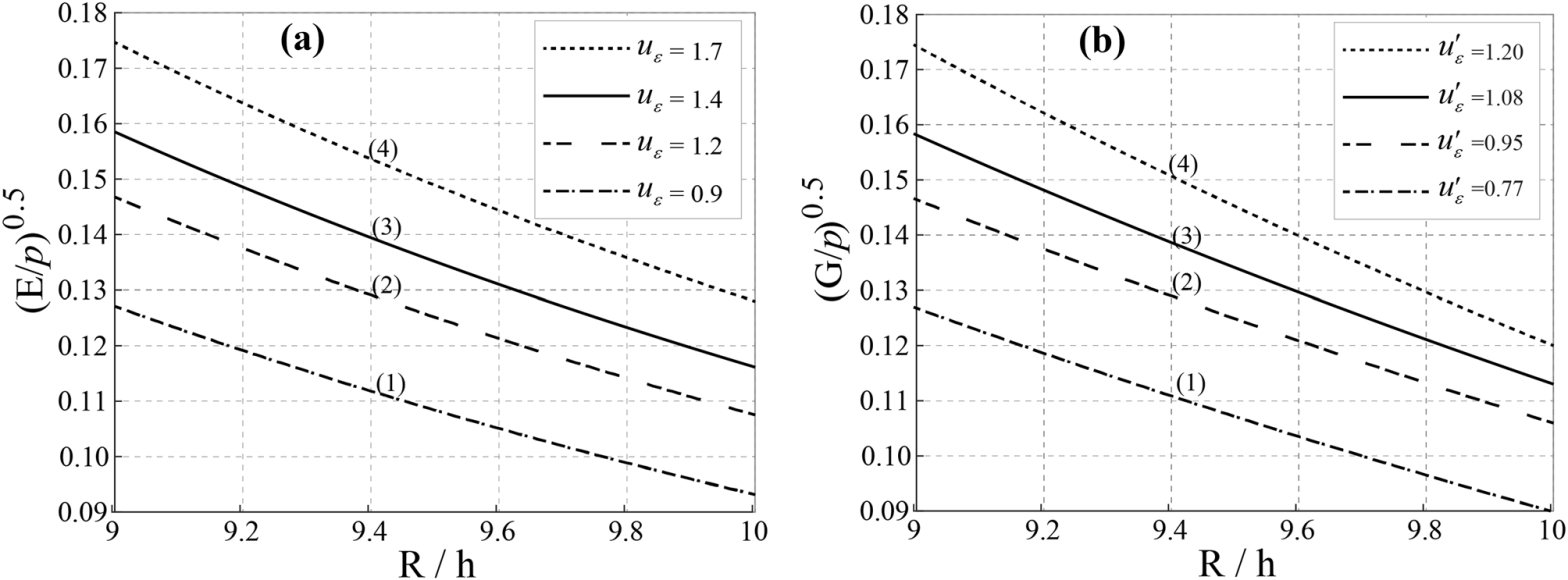
Displacement induces by the ground vibration. (**a**) *u*_ε_ (Initial flow velocity (m/s)), and (**b**) *u*'_ε_ (Propagation velocity (m/s)).

Figure 9a, b demonstrate the relationship between *u*_ε_ and $u^{\prime}_{\varepsilon }$. The displacement curves exhibit a ratio of *R*/*h* = 0.5, with *u*_ε_ varying between 0.25 m and 0.35 m and $u^{\prime}_{\varepsilon }$ ranging between 0.225 m and 0.288 m. Additionally, Figs. 10 show that *u*_z_ has a vertical displacement within a range of *R*/*h* between 9 and 10. The values for vertical displacement are categorized as follows: (1) 0.092–0.13 m, (2) 0.11–0.15 m, (3) 0.12–0.16 m, and (4) 0.13–0.18 m in Fig. 10a, and (1) 0.09–0.129 m, (2) 0.118–0.146 m, (3) 0.115–0.159 m, and (4) 0.12–0.175 m in Fig. 10b**.** Soil porosity and depth significantly influence fluid infiltration and ground vibrations in deep coal mines. As depth (*h*) increases, the ground vibration index decreases due to greater compressibility and consolidation of strata, leading to reduced displacement and permeability (*K*_c_). Non-homogeneous soil complicates displacement patterns, with deeper layers exhibiting lower displacement due to dampening effects, impacting stability and water loss assessments. The relationship between vertical displacement and radial distance from the central axis indicates that vibrations diminish with depth, facilitating efficient coal extraction. However, intense vibrations can cause stress variations, potentially resulting in settlement within the mine.

##### Monitoring and mitigation strategies for water loss

To mitigate the impact of fractures and vibrations on groundwater dynamics, real-time monitoring and mitigation strategies are essential. Table 8 highlight the relationship between drawdown (*Dd*), well discharge (*w*), and soil porosity (*ϕ*). Drawdown ranges from *-*0.1 to 0.066, while permeability constants average 0.007.

**Table 8:** Variables related to infiltration and water protection.

Flux	Time (t (s))										
	0	1	2	3	4	5	6	7	8	9	10
*Dd*	0	−0.13	0.07	0.04	0.03	0.03	0.02	0.02	0.02	0.01	0.01
*w*	0	0.003	0.003	0.003	0.003	0.003	0.003	0.003	0.003	0.003	0.003
*ϕ*	0	−0.001	−0.11	−0.03	−0.02	−0.01	−0.01	−0.003	−0.002	−0.002	−0.001
*q (*z)	0	−0.003	−0.75	−0.37	−0.22	−0.15	−0.11	−0.09	−0.07	−0.06	−0.05

q is the Darcy flux (m/s) ; ϕ is the soil porosity (%); w is the well discharge (m^3^/s), Dd is the drawdown.

The total flow rate over 10 s decreases from 0.033 *L*/*s* to 0.0034 *L*/*s*, with *ρ* = 1.92 m^3^/s. Hence, permeability coefficients (*K*_c_) vary from 0.027 to 0.003. Effective monitoring of these parameters ensures groundwater stability, reducing risks associated with mining-induced vibrations and improving water management strategies.

**Fig. 11:**
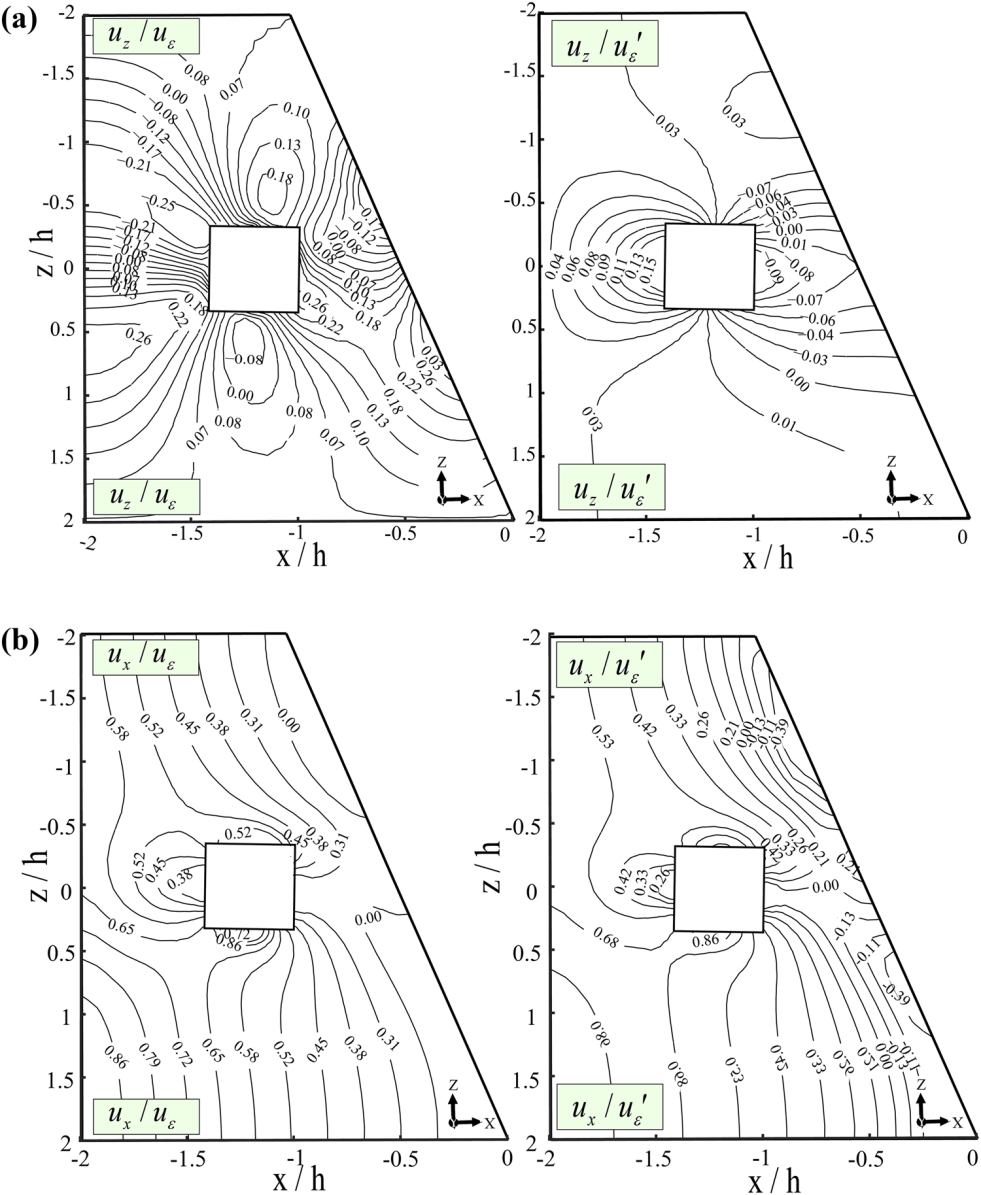
Contour Lines Illustrating Groundwater Movement axis. (**a**) Vertical displacement (*u*_z_), and (**b**) Horizontal displacement (*u*_x_).

Figure 11a, b illustrate contour lines representing groundwater movement around the working space. These graphical representations depict groundwater displacement along fractures converging toward the mining area. The irregularity of the mine walls contributes to variations in the contour lines, reflecting both horizontal and vertical displacements along the inner surface. The contour lines are concentric, with horizontal displacement (*u*_x_) values ranging from 0.00 to 0.86 and vertical displacement (*u*_z_) varying between -0.25 and 0.18. These variations highlight the influence of structural irregularities on groundwater flow dynamics within the mine. The fluid infiltration and water balance analysis should incorporate hydrogeological mapping to identify critical fracture zones affecting water retention. Seepage control systems, such as grouting, can seal high-permeability fractures, while vibration dampening measures help minimize stress-induced permeability changes. Continuous water-level monitoring with piezometers ensures real-time detection of groundwater fluctuations, enhancing water management. This section highlights the interconnected relationship between fractures, permeability loss, vibrations, and groundwater dynamics in the Datong Mine. By implementing targeted monitoring and mitigation strategies, mining operations can minimize environmental and operational risks associated with groundwater depletion.

### Field-based validation of stress and groundwater modeling

#### Field data for model validation

Rock mechanics tests on Seam No. 12 samples validate the numerical model by assessing mechanical properties. Uniaxial compression tests show coal has a compressive strength of 22.89 MPa and elasticity modulus of 4.01 GPa, while rock exhibits 64.30 MPa and 33.10 GPa, respectively. Tensile strength and Poisson ratio are 2.09 MPa, 0.27 (coal) and 6.47 MPa, 0.22 (rock). These values serve as input for finite element simulations to ensure accurate stress modeling. Vibration monitoring near the 5302 working face uses accelerometers and geophones to assess ground motion. The maximum stress concentration reaches 2.8 MPa, significantly affecting stability. The ground vibration index (*I*v) reaches 0.005 m, five times the threshold of 0.001 m, indicating severe dynamic stress from vibrations. As discussed in Sect. 3.2, permeability decreases significantly, while water infiltration increases by 0.11 m per fracture, highlighting the link between mining operations and groundwater loss.

Hydrogeological monitoring with piezometers and flow meters analyzes groundwater flow. Aquifer permeability significantly decreases, and vertical displacement reaches up to 0.18 m in high-porosity zones (*ϕ* ≈ 0.25). Dewatering efficiency improves, with external pumping at 0.31 m^3^/s and internal at 16.43 m^3^/s, ensuring mine stability. These results confirm the model’s assumptions, demonstrating the correlation between stress redistribution, permeability changes, and groundwater depletion.

#### Settlement of the upper space induced by vibrations

**Fig. 12:**
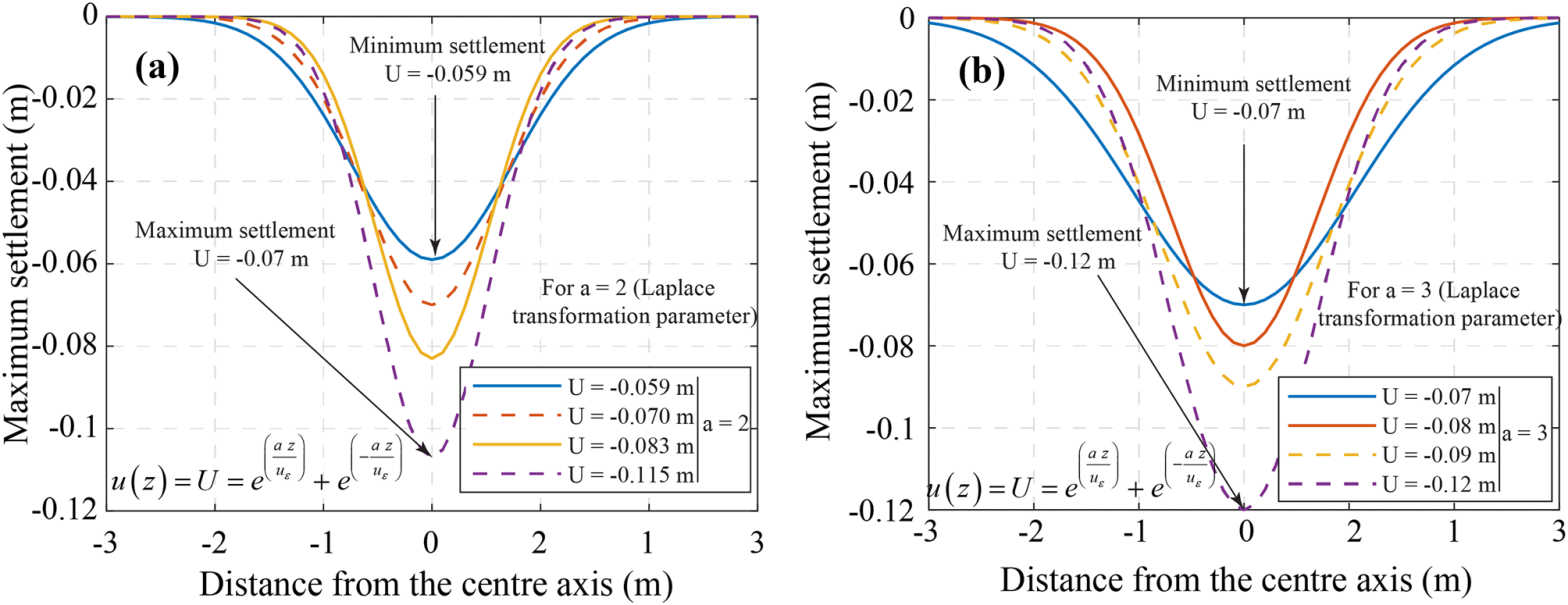
Ground settlement induces based on the Laplace transformation parameter. (**a**) a = 2, and (**b**) a = 3.

**Fig. 13:**
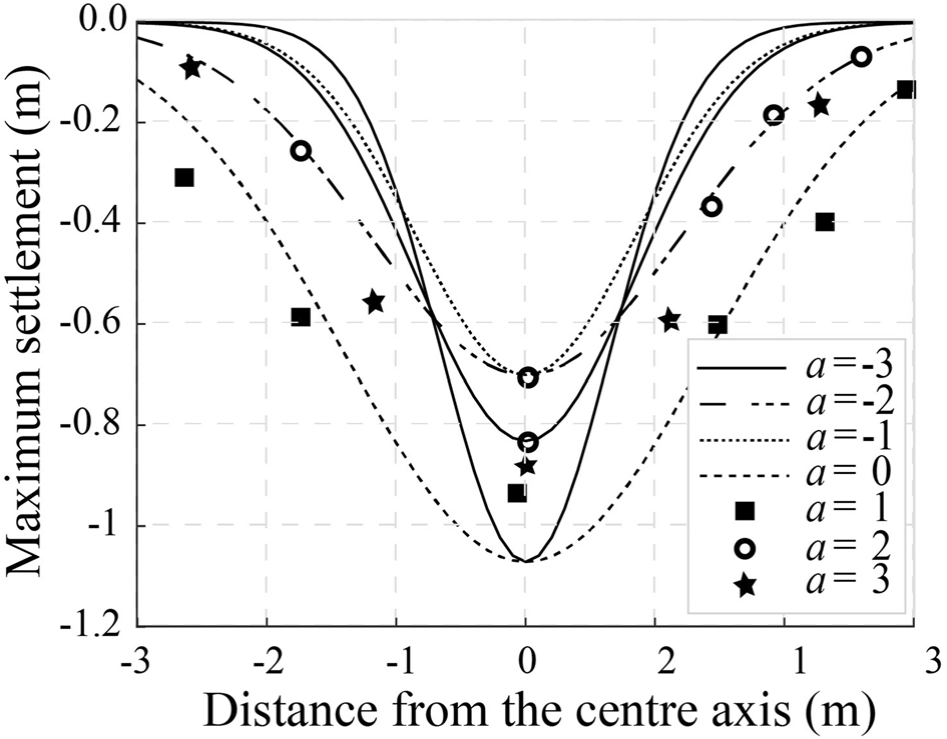
Ground settlement for $\overline{u}_{z}$/*u*_*ε*_, with −3 < *a* < 3.

Accurate prediction of fault depth and orientation is crucial for mitigating water damage in coal seams^33^. Figure 12a, b illustrate vertical settlement due to internal soil pressure reduction. As *a* = 2, settlement (*U*) ranges from **−**0.06 m to −0.105 m**,** while for *a* = 3, it increases to −0.07 m to −0.12 m. This indicates intensified soil compaction, impacting mine stability and groundwater flow. Figure 13 presents a settlement trough (*a* = *−*3 to 3), derived using Fourier transformations. Scalar values (*u*_ε_ = 1.70 m, 1.42 m, 1.20 m, 0.93 m) highlight broader settlement under complex loading. These results enhance the accuracy of settlement predictions, as the data consistently meet the benchmark of a minimum settlement of 0.12 m. Thus, the redistribution of stresses and the control of excavation guarantee structural integrity and groundwater management in mining operations.

### Ground vibrations on soil displacement and fluid infiltration analysis

#### Integrated analysis of stress, strain, and vibrations in Datong

Groundwater loss in the Datong coal mine is modeled through a multi-scale approach, integrating stress concentration gradients, strain rates, and material properties. Using Fourier’s heat conduction and Cauchy’s momentum equations, microscopic analyses assess fracture propagation and permeability reduction under vibrations exceeding 6 MPa. Macroscopic studies examine regional groundwater depletion, validated by ABAQUS simulations and the Hoek–Brown criterion. This framework harmonizes geomechanical and hydrogeological assessments, improving stability predictions and environmental mitigation. By capturing both local excavation impacts and broader hydrological disruptions, the model provides a comprehensive strategy for groundwater management in deep coal mining.

#### Time-dependent evolution of permeability

The simulation results show that aquifer permeability evolves dynamically during the loading period. Initially, the development and expansion of fractures due to vibration-induced stress lead to a measurable increase in permeability up to approximately 15–25% above baseline values. However, with continued loading, settlement of overburden layers and migration of fine particles into newly formed pores progressively reduce permeability. As discussed in Sect. 3.2, permeability declines by approximately 60% over the simulation period, primarily due to cumulative stress redistribution, fracture closure, and fluid-rock interactions. This transient behavior underscores the importance of time-resolved modeling to accurately capture aquifer responses in dynamic mining environments.

#### Fluid infiltration patterns and groundwater loss analysis

**Fig. 14:**
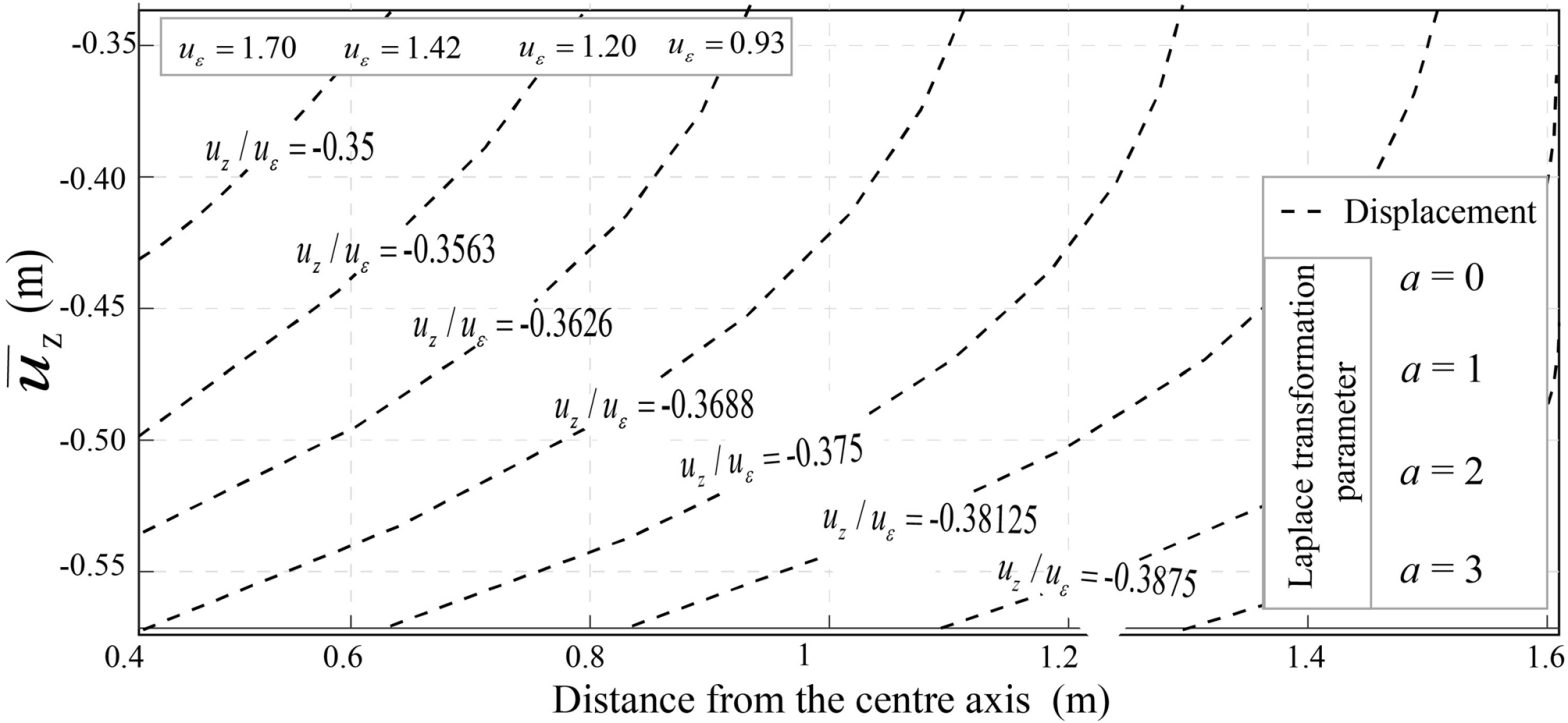
Derivation of parameter for the vertical displacement conjugates $\overline{u}_{z} /u_{\varepsilon }$ for $a \ge 0$.

**Fig. 15:**
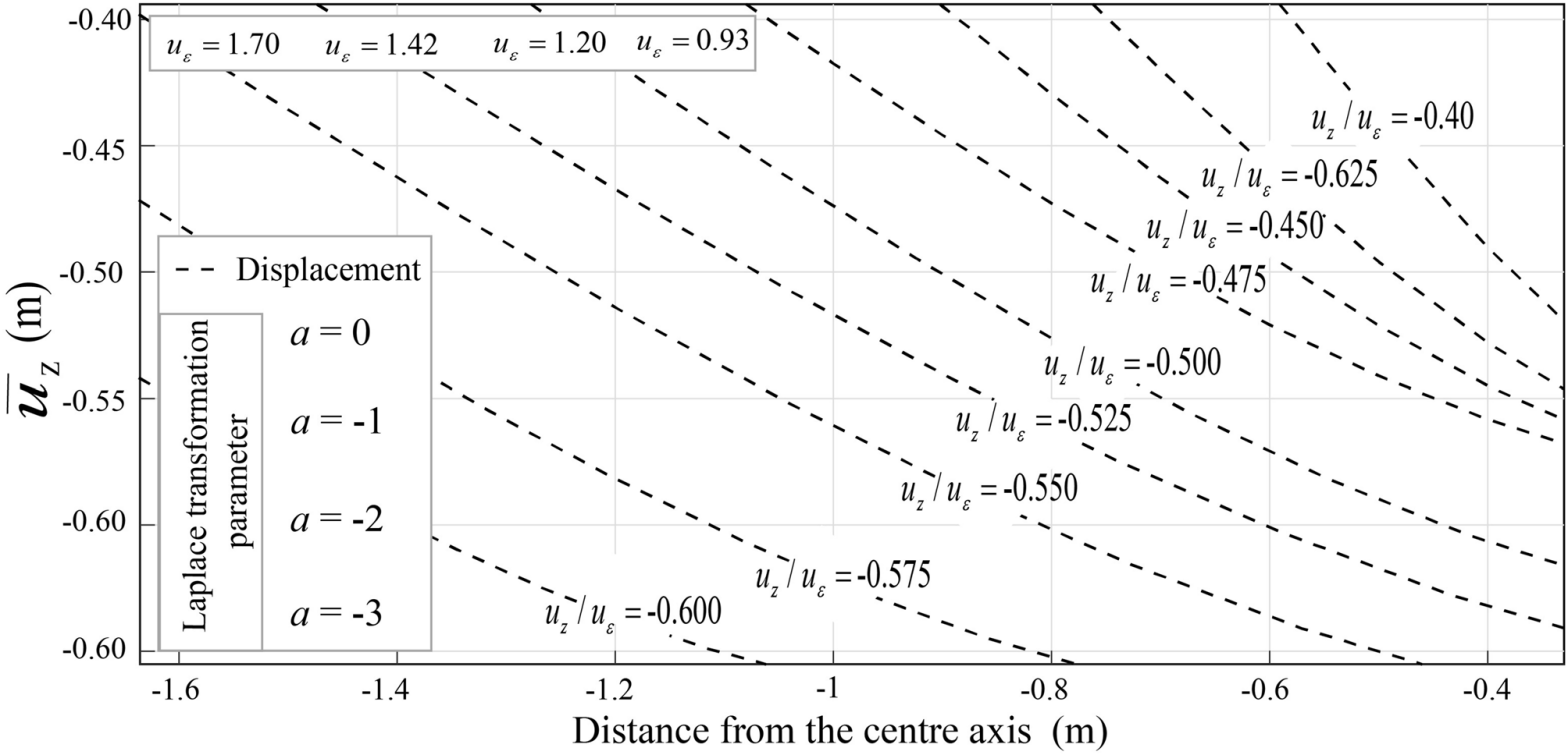
Derivation of parameter for the vertical displacement $\overline{u}_{z} /u_{\varepsilon }$ for $a \le 0$.

Groundwater dynamics are deeply influenced by soil vibrations, as demonstrated through analytical solutions. Parameters such as porosity (*ϕ*), well discharge (*w*), drawdown (*Dd*), and displacements (*u*_x_, *u*_z_) highlight the mechanical-hydrogeological interplay. A space–time coupling equation incorporating infiltration rate, internal friction angle (*φ*), and density of water (*ρ*) predicts rock instability in coal seams, including vein No. 12. For unsaturated soils (*u*_ε_ = 0.56 m), Table 9 reveals Transmissivity ranging from 0 to 51.65, Storativity from 0 to 108.68, and flow rates peaking at 15.52. Vertical displacements converge towards *R*/*h* = 0 due to transverse tunnel pressure reductions, ensuring improved excavation stability (Figs. 14, 15).

**Table 9:** Storativity, Transmissivity, and average water flow.

Time(s)	0	1	2	3	4	5	6	7	8	9	10
T	0	−2.4e−4	18.86	23.62	26.20	28.92	34.75	37.87	41.13	48.00	51.65
S	0	−0.7e−4	7.94	14. 91	22.05	30.42	43.87	55.78	69.23	90.91	108.68
Q	0	5.7e−4	15.52	12.89	10.70	9.43	9.414	8.78	8.34	8.63	8.34

T is the Transmissivity; S is the Storativity, and Q is the average water flow.

**Fig. 16:**
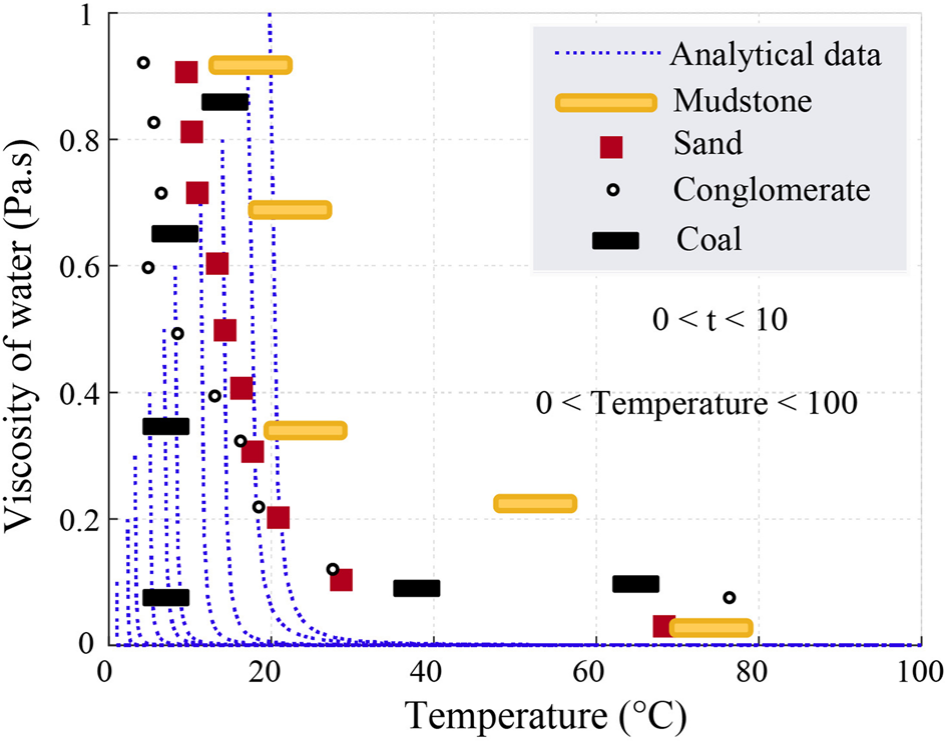
Dynamic viscosity of water.

Time-dependent flow rates decrease consistently, highlighting the temporal nature of hydrogeological adjustments in response to vibration-induced stress. These findings underscore the necessity of accounting for soil porosity and permeability in managing groundwater behavior in areas affected by ground vibrations. The viscosity of water (*μ*), as illustrated in Fig. 16, demonstrates a temperature-dependent decline from 1 Pa.s at 0 °C to 0 Pa.s at 100 °C. Thermodynamic parameters such as void ratio (*e*_*v*_), volume of the solid (*V*_*s*_), and total volume of water (*V*_*T*_) were calculated, yielding averages of *V*_*v*_ = 3.e^-5^ m3, *V*_*s*_ = 1.6 m^3^, and *V*_*T*_ = 1.55 m^3^. These results reveal the interdependence between water properties and soil vibrations, further emphasizing the need to integrate thermal and mechanical factors into groundwater models.

The geological analysis of the mine identifies soil vibrations as a key driver of groundwater depletion, influenced by fault orientation, stress asymmetry, and subsurface cracks. Vibrations disrupt stress distribution, increase water pressure, and alter drainage, with thermal factors and porosity dynamics further complicating water flow. Therefore, the mitigating this impact requires integrated management strategies addressing geological and hydrogeological interactions. Proactive approaches, including stress redistribution and fault stabilization, are essential to minimize vibration-induced water loss. The study underscores the need for sustainable practices to protect hydrogeological systems in mining environments and areas prone to soil vibrations.

### Numerical simulation and coupling mechanisms of ground vibrations

Finite Element Modelling (FEM) and Discrete Element Modelling (DEM) are fundamental tools for analyzing ground vibrations and mining-induced stress alterations. In particular, FEM, implemented via Abaqus, effectively simulates stress accumulation and redistribution within rock masses, while DEM captures granular interactions and the initiation of rock fractures. Building on these capabilities, the hybrid modeling framework developed by Omar et al.^34^, which integrates the Analytic Element Method (AEM) with the Finite Difference Method (FDM) offers a transferable approach well-suited for flood-prone alluvial environments such as the Kulsi River Basin. This integrated methodology enhances the evaluation of flood-induced groundwater recharge and facilitates a more accurate simulation of hydrological connectivity. Furthermore, the adoption of multi-layered and transient calibration strategies, as demonstrated in their study, strengthens model reliability and predictive performance. Applying these insights to the Datong mine context, simulation results indicate that stress concentration zones significantly influence groundwater flow patterns and permeability, promoting settlement and water infiltration.

**Fig. 17:**
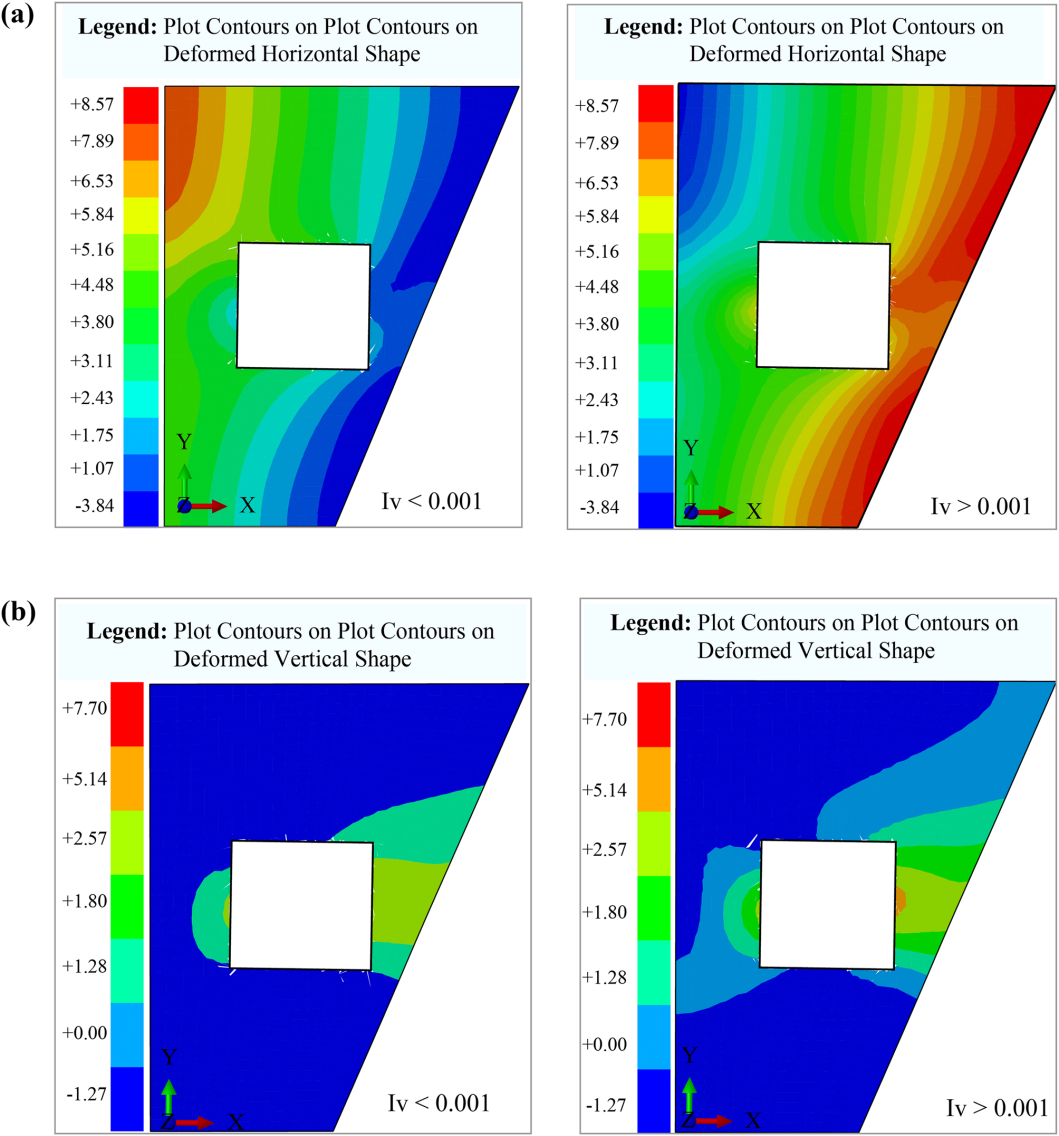
Numerical simulation of horizontal and vertical displacement. (**a**) Mine geometry and R/h values (Technical comment: illustrates the effect of R/h values on soil deformation during excavation), and (**b**) Soil deformation based on R/h values (Technical comment: shows the displacement of ground layers and water infiltration intensity during excavation)

Figure 17 illustrates numerical simulations of horizontal and vertical displacements in the coal mine, utilizing the mechanical and physical properties of the geological materials. The model integrates Fourier’s law and Cauchy’s equation. ABAQUS simulations validated its accuracy, showing the finite element analysis software renowned for its accuracy in modeling complex soil-structure interactions. Key input parameters include the bulk modulus (*K* = 1.3 MPa), shear modulus (*G* = 2000 MPa), soil cohesion (*C* = 1 MPa), and internal friction angle (*φ* = 20∘). The cohesion value used here corresponds to the effective in-situ condition of the fractured coal mass and was chosen to realistically simulate the mechanical response under dynamic loading. Although this is lower than typical values for intact coal, it is consistent with observed field behavior and was validated during model calibration. The tensile strength, calculated as 0.3 MPa, reflects the characteristics of Coal Seam No. 12, enabling precise representation of the stress–strain behavior in this heterogeneous environment.

Figure 17a, b present numerical simulations of horizontal and vertical displacements in the Datong coal mine using ABAQUS. Figure 17a highlights horizontal mesh deformations, showing stress wave propagation and high-pressure zones, while Fig. 17b illustrates vertical displacement trends (ranging from 0.02 to −0.09), and indicating settlement and infiltration effects. As developed by the Drucker-Prager model, the models incorporate bulk modulus (1.3 MPa), shear modulus (2000 MPa), soil cohesion (1 MPa), internal friction angle (20°), and tensile strength (0.3 MPa). Enhancing color gradients for readability and using standardized geological symbols for fractures, stress concentrations, and high-pressure zones improve interpretation and application in mining engineering. The results strongly align with theoretical predictions, providing a robust framework for evaluating ground stability in mining operations.

The findings corroborate Senjuntichai et al.^35^, who highlighted the influence of vibratory excitation frequency, foundation flexibility, and embedment on saturated soil dynamics, emphasizing the role of soil permeability and geometric factors. Similarly, they align with prior research^36^ on strata pressure redistribution in extra-thick coal seams, validating the numerical framework. Integrating material properties, geometrical factors, and vibratory dynamics enhances predictive accuracy and supports effective risk mitigation. The study advances mining safety and sustainability practices by offering insights into the interplay of stress redistribution, deformation mechanisms, and subsurface fluid dynamics. These models illustrate vibration propagation through heterogeneous rock masses, improving predictive accuracy and supporting sustainable mining strategies to mitigate water loss and ground instability. To extend the analytical framework, exploring software interoperability, particularly between the Hydrologic Engineering Center’s River Analysis System and groundwater models such as the Modular Finite-Difference Ground-Water Flow Model is recommended to support a more integrated and comprehensive water resource management strategy.

### Drucker-Prager model application and validation

Laboratory tests on Datong mine samples provided key parameters, including a shear modulus of 2000 MPa, cohesion of 1 MPa, and a friction angle of 20°. These values were validated through triaxial compression tests and Abaqus simulations to ensure their alignment with observed stress–strain behaviors. The mechanical properties used in the simulations confirmed their alignment with field measurements.

A numerical sensitivity analysis refined the model, testing variations in cohesion, bulk modulus, and friction angle. The Drucker-Prager model effectively captured stress redistribution, deformation, and vibratory effects, aligning with field data. Its validated performance confirms its suitability for modeling fractured coal and rock masses under dynamic loading, supporting accurate predictions for stress propagation and settlement in mining operations. Figure 17 (Numerical simulation of displacement) validates the Drucker-Prager model through Abaqus-based finite element analysis.

### Experimental validation of ground vibrations in Datong

This study explores advanced techniques for assessing mine stability beneath inclined planes, focusing on practical engineering applications. The Datong coal mine, with recoverable reserves below 800 m, includes Seam No. 12, which spans 50 km and averages 23.81 m in thickness. Tracer tests combining temperature variations and water transport dynamics evaluate deformation caused by infiltration during excavation.

**Fig. 18:**
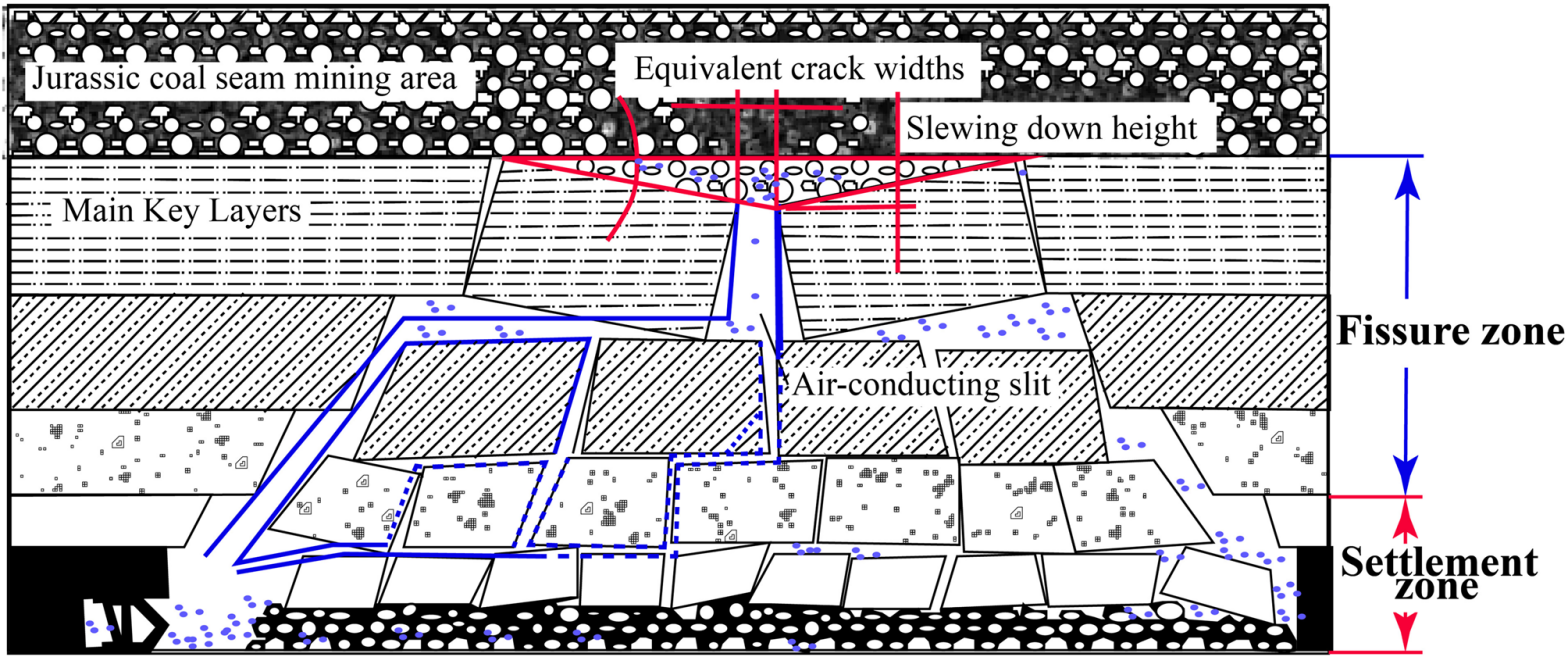
Classification of Overlying Strata into Fracture and Settlement Zones Based on Hydraulic Conductivity.

**Fig. 19:**
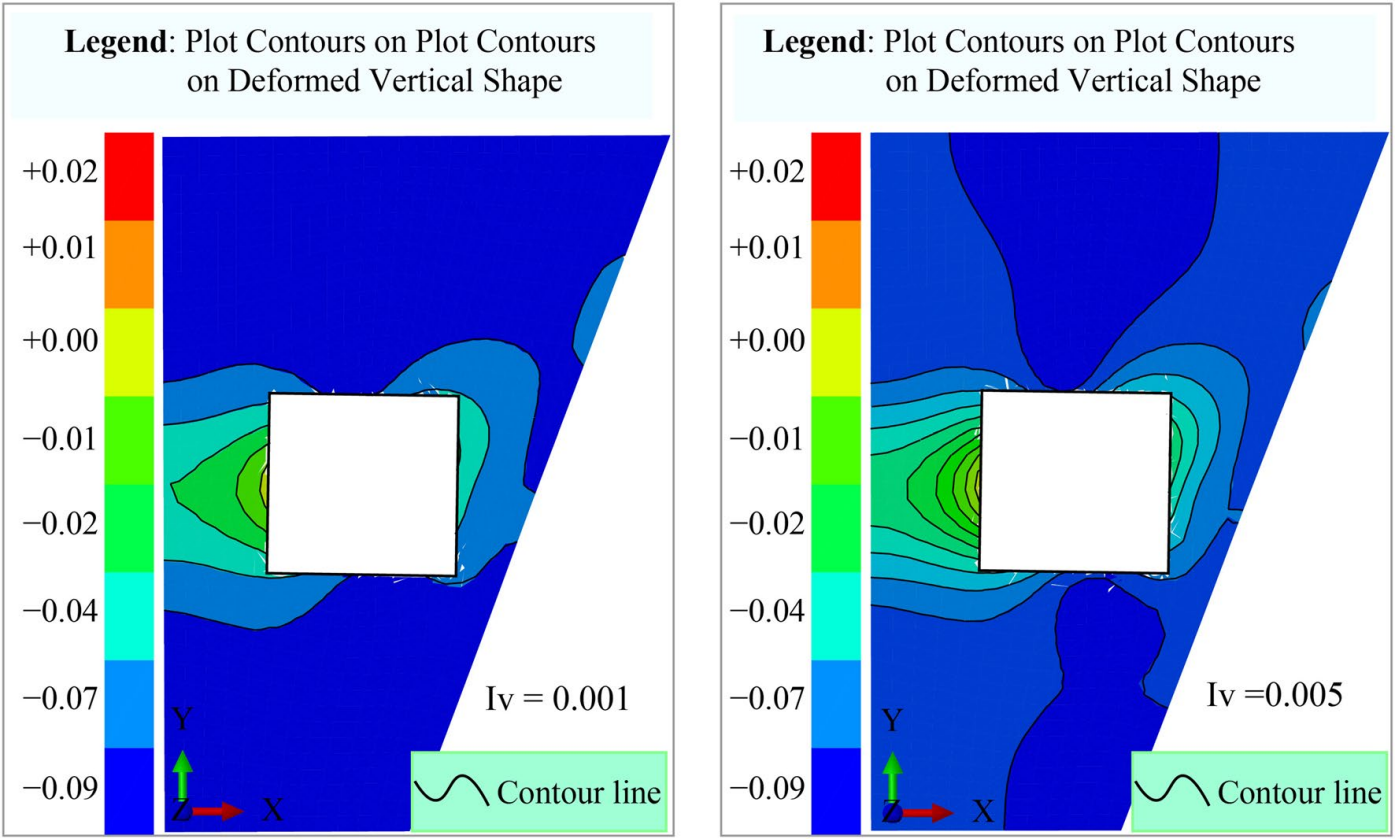
Multi-Physics Simulation of Fluid Dynamics and Temperature Variations in Coal Strata.

To analyze groundwater release in Jurassic goafs, a discriminant process was established to assess whether groundwater could enter the working space of the Carboniferous coal seam. Four parameters equivalent fracture width, equivalent fracture length, equivalent fracture channel length, and pressure difference between flows fields were selected to develop a multi-parameter control equation. The equivalent fracture width, which impacts groundwater transport, is simplified to a main fracture equating to the total volume of smaller fractures. The equivalent cross-section of the groundwater escape channel is illustrated in Fig. 18. Fourier series-based Laplace transforms highlight mine disturbances, revealing stress and shear variations exceeding 6 MPa. Fluid dynamics and porosity are simulated using Darcy’s law and Navier–Stokes equations, with OpenPLAM multi-physics results demonstrating turbulent dynamics and temperature effects. The multi-physics simulation indicates a hydraulic conductivity (*C*_h_) of approximately 0.005 m/s, with soil porosity (*ϕ*) values ranging from 0.2 to 0.3. It’s crucial to consider the insights gained from Fig. 19, which illustrates the impacts of coal seam burial depths and return flows on water dynamics. The graph displays groundwater behavior across varying depths (83–191 m) and thickness (7.5–9.1 m, averaging 8.3 m), providing a visual representation of how these factors influence water flow and stability within mining environments.

During mining operation, continuous monitoring with inclinometers and piezometers detect ground motion and water pore pressure changes, predicting potential instability. Effective groundwater management is essential to mitigate risks and optimize operations. Dewatering systems and drainage strategies reduce water loss and enhance safety, achieving average external and internal pumping rates of 0.31 m^3^/s and 16.43 m^3^/s, respectively. These measures improve operational resilience and ensure sustainable mining practices.

The method demonstrates notable improvements compared to previous studies^37,38^. The confining pressure for coal samples from Seam No. 12 is measured at 0.32 MPa, which is consistent with values for granitic soils, underscoring the method’s reliability. It addresses long-term environmental impacts, including groundwater depletion and potential contamination, highlighting the need for sustainable management to mitigate irreversible effects. By integrating detailed simulations, innovative testing, and robust management strategies, the study enhances the practical applicability of these methods, ensuring improved stability and environmental sustainability in mining operations.

## Discussion

### Dynamic permeability behavior

**Fig. 20:**
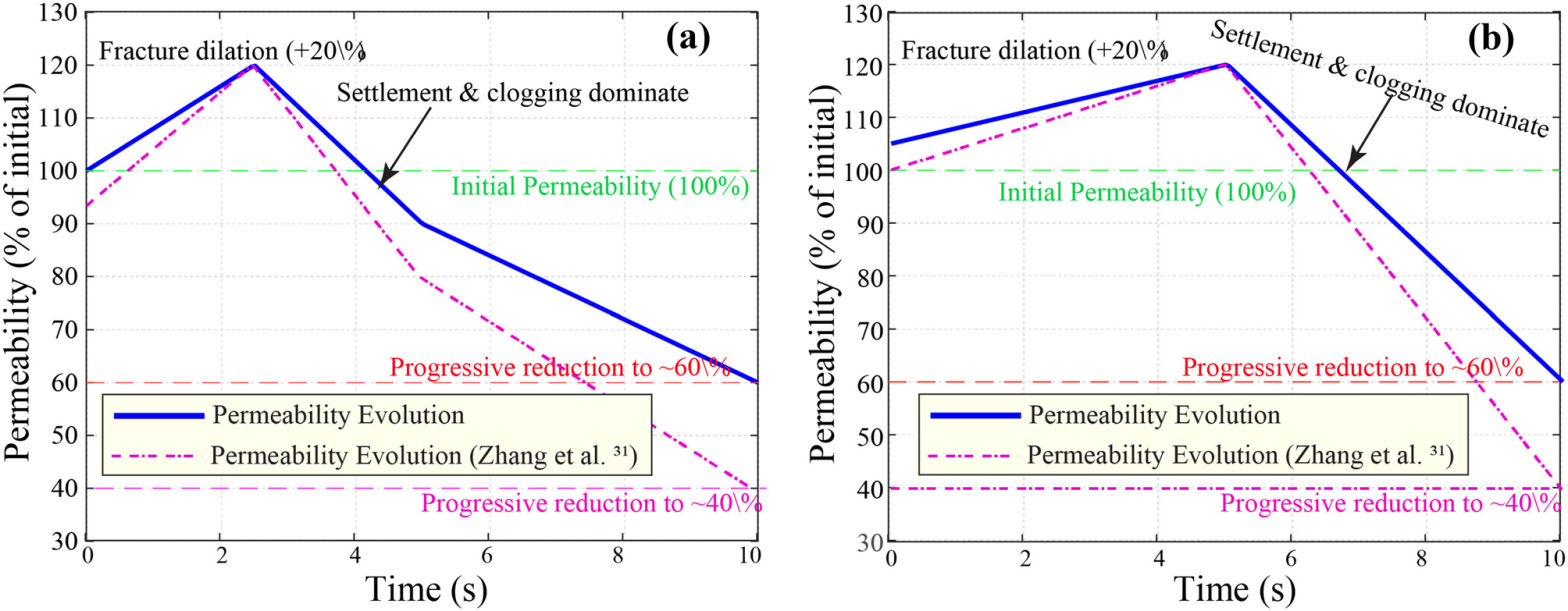
Time-Dependent Evolution of Permeability.

The study reveals dynamic aquifer permeability under vibration stress exhibits time-dependent contrasts: an initial 15–25% rise (0–5 s) from fracture dilation, followed by progressive clogging (5–10 s), culminating in a net 60% reduction (*t* = 10 s; Fig. 20). This exceeds the 40% static-load reduction reported by Zhang et al.^31^, highlighting dynamic stress’s amplified transient fracture connectivity before long-term clogging dominates. Balcha et al.^5^ observed vibration-induced compaction but lacked porosity thresholds (Sect. 3.2). The 60% loss underscores hydro-mechanical interplay where short-term permeability gains (0–5 s) are eclipsed by particle migration and overburden settlement (5–10 s). Compared to steady-state models, dynamic loading induces a 20% greater decline, emphasizing temporal evolution’s critical role in predicting mining-induced water inflow.

### Ground vibrations and groundwater loss in mining

Recent advances in groundwater modeling have highlighted the value of scenario-based, three-dimensional (3D) numerical simulations for capturing complex flow dynamics in heterogeneous subsurface environments. Omar et al.^39^ proposed a modular 3D numerical framework to model groundwater flow under various geological and operational conditions, demonstrating its flexibility and accuracy in predicting aquifer responses.

The results of this study indicate a significant relationship between ground vibrations and groundwater loss, particularly with an observed average vibration index of *I*v = 0.005 m, which is approximately five times the normal vibration index of *I*v = 0.001 m. This correlation is visually represented in the “Displacement Induced by Ground Vibration” figure, illustrating how increased vibrations lead to substantial displacements and stress concentrations in the surrounding geological formations. While numerical models offer high spatial resolution and adaptability, they are often computationally intensive and data-dependent. In contrast, the analytical model developed in this study provides a simplified yet effective means of estimating groundwater loss induced by dynamic ground vibrations. This finding aligns with Zhang et al.^31^, who showed that elevated ground vibrations compromise structural integrity and increase permeability, altering groundwater flow.

Additionally, the study’s findings regarding an average water infiltration distance of 0.11 m between cracks align with previous conclusions^40^. Mining-induced vibrations significantly alter groundwater flow paths, resulting in increased water loss through fractures. Comparing these findings highlight the urgent need for effective monitoring and management strategies to mitigate the adverse effects of mining-induced vibrations on groundwater resources. This underscores the importance of integrating analytical models with empirical data to promote sustainable mining practices.

Field studies and controlled laboratory experiments in the Datong coal mine examined the effects of mining-induced vibrations on the surrounding rock and groundwater. Vibration monitoring using accelerometers and geophones measured stress concentrations, with maximum values reaching 2.8 MPa, significantly affecting stability. Key parameters such as pore pressure changes, permeability reductions, and groundwater flow alterations were evaluated. As discussed in Sect. 3.2, and 3.3 permeability significantly declines during the simulation period, while vertical displacement peaks at 0.18 m in high-porosity zones (ϕ ≈ 0.25), measured under vibratory stress of 6 MPa (Table 10). Hydrogeological monitoring using piezometers and flow meters confirms the correlation between vibration intensity, stress redistribution, and aquifer depletion, thereby validating the numerical modeling predictions.

**Table 10:** Summary of groundwater loss drivers.

No	Parameter	Value	Impact
1	Critical stress	6 MPa	Triggers fracture propagation
2	Peak displacement	0.18 m	Correlates with ϕ > 0.25 zones
3	Net permeability	60%	Settling + particle clogging
4	Infiltration rate	0.11 m/ fracture	Increases water Loss risk

The analytical model is based on assumptions of linear poroelastic behavior, fracture-dominated flow, and uniform stress wave propagation, making it suitable for moderately permeable fractured formations. However, in highly plastic clays, non-linear, time-dependent viscoplastic behavior and low hydraulic conductivity limit its accuracy, as groundwater migration is diffusion-dominated and transient permeability changes are minimal. In competent, unfractured rocks, limited fracture connectivity reduces the effect of dynamic loading on groundwater movement, leading to over- or underestimation. To improve applicability, future models should integrate site-specific mechanical and hydraulic properties or hybrid approaches capturing non-linear, anisotropic, and time-dependent geological behaviors.

## Conclusions

This study develops an analytical model to predict groundwater loss in deep coal mines, focusing on the effects of ground vibrations. The research objective is to enhance the predictive accuracy of dynamic stress from vibration distributions and hydrogeological changes to mitigate water loss during mining operations. Laboratory tests using Datong mine samples reveal critical physico-mechanical parameters, including soil cohesion (1 MPa), Poisson ratio (0.35), and effective elastic modulus (12.5 GPa), which are used to validate the model. The model adopts an effective elastic modulus of 12.5 GPa to represent the fractured and compositionally mixed coal-rock zone. Although the measured elastic moduli of coal and rock are 4.01 GPa and 33.10 GPa respectively, the adopted effective value of 12.5 GPa more accurately reflects the in-situ mechanical behavior of the fractured and vibration-affected coal-rock mass. While the simulation parameters differ from laboratory values for intact materials, the use of calibrated, field-representative properties enabled the model to reproduce observed groundwater flow patterns and deformation characteristics. This approach ensures consistency between numerical predictions and actual field behavior, enhancing the model’s practical reliability.

Stress concentration zones exhibit maximum vertical stress values of 2.8 MPa, correlating with a 60% reduction in aquifer permeability and significant shifts in water retention capacity. The observed reduction in aquifer permeability reflects the net outcome of counteracting processes: an initial, transient rise linked to fracture formation, ultimately overshadowed by a sustained decrease driven by stress-mediated compaction and pore occlusion. This recognition of phased dynamics underscores the model’s predictive reliability and validates its application in dynamic subsurface environments subject to vibrational stresses. This methodology extends to other mining environments, including open-pit and gold mining operations, where similar vibrational stresses influence hydrogeological systems. Its application in geotechnical monitoring enhances water retention strategies and operational efficiency, offering a robust framework for minimizing environmental impacts.

The study demonstrates that vibration-induced fractures in coal and rock strata fracture alter groundwater flow dynamics, leading to increased infiltration rates. The model quantifies infiltration distances, which average 0.11 m per fracture under vibratory stress conditions. Vertical displacement patterns peak at 0.18 m, primarily in zones characterized by high porosity (*ϕ* ≈ 0.25) and moderate transmissivity (*T* = 0.01 m^2^/s). Temporal flow rates decrease progressively, reflecting the transient nature of stress redistribution and its influence on aquifer behavior. These findings confirm that the interaction between ground vibrations and subsurface structures significantly impacts water flow, creating complex challenges for groundwater management.

By integrating theoretical and empirical insights, this study provides a practical approach to sustainable mining practices. It emphasizes the importance of incorporating stress-induced hydrogeological modeling into mine planning to mitigate the adverse effects of mining-induced vibrations. Additionally, the model facilitates improved groundwater resource management, ensuring the long-term viability aquifers in heavily exploited regions.

This work underscores the importance of predictive models in addressing water loss issues in mining. It advocates for the proactive integration of hydrogeological strategies in resource extraction to protect environmental resources and enhance the sustainability of mining operations. Future developments aim to incorporate anisotropic soil behaviors and thermal–hydraulic interactions into the model to expand its relevance across diverse geological settings.

## Supplementary Information

Supplementary Information.

## Data Availability

All data, models, and code generated or used during the study appear in the submitted article.
