# Supplementary material for: Analytical prediction of groundwater loss in deep coal mines induced by ground vibration
Source: Sci Rep. 2025 Jul 22;15:26578. doi: 10.1038/s41598-025-05970-6 (PMC12284197; doi:10.1038/s41598-025-05970-6)
Supplement: Supplementary file 1 — Supplementary Information. [file 41598_2025_5970_MOESM1_ESM.docx]

# Appendices

## A.1 Terminology Related to Groundwater Dynamics and Mining

Coal Mining Terminology: Defining Key Concepts for Clarity and Precision.

**Caved Zone**: This typically refers to an area where the roof of a mine has collapsed, often due to the removal of coal or other materials. This zone can impact both stability and groundwater flow patterns.

**Ground vibrations:** Subsurface oscillations induced by mining

**Fractured Zone**: This term describes an area within the subsurface where the rock or coal strata are broken into fragments, typically leading to altered hydraulic properties. These zones often influence water movement and storage capacity.

**Aquifer Permeability**: Refers to the ability of a rock formation to transmit water through its pore spaces and fractures. In mining contexts, understanding changes in permeability due to mining activities is critical.

**Vibrational Loads**: These are stresses exerted on the geological strata as a result of mining activities (e.g., blasting or equipment operation), which can lead to seismic effects and hence influence groundwater dynamics.

**Hydrogeological Dynamics**: This term describes the movement and distribution of groundwater and its relationship with geological formations. In mining, changes can occur due to excavation or induced vibrations that alter flow paths.

**Porosity**: The measure of void spaces in materials, which is crucial for determining how much water can be stored within rock or soil.

**Transmissivity**: A measure of how much water can be transmitted horizontally through an aquifer, significant in assessing the flow potential in fractured or caved zones.

**Ground settlement:** The downward movement or settling of the Earth's surface resulting from the removal of subsurface material a frequent consequence of coal mining operations.

**Goaf**: Mined-out void area

## A.2 List of Notations

| Symbol |  | Meaning | Symbol |  | Meaning |
| --- | --- | --- | --- | --- | --- |
| $\sigma_{x},\sigma_{z}$ | : | Horizontal and vertical stress (MPa) | $\bar{u}\left(z,a\right)$ | : | Displacement conjugation (m) |
| *a* |  | Fourier series parameter or a Laplace transform coefficient | w |  | Well discharge (m³/s) |
| b | : | Thickness of the aquifer (m) | *γ_ω_* |  | Volumetric weight of the fluid (m³) |
| *C*_h_ | : | Hydraulic conductivity (m/s) | Q |  | Flow rate or average water flow (m³/s) |
| Dd |  | Drawdown (m) | F | : | Inertial force of the solid (MPa) |
| *D_i_* | : | Discharge rate (m³/s) | $v$ | : | Poisson ratio |
| *g_0_* | : | Gravitational charge (kg) | q (z) | : | Darcy flux (m/s) |
| H | : | Heaviside unit step function | $\omega_{v}$ | : | Frequency of the vibration (Hz) |
| h | : | Depth (m) | u’_0_ | : | Vibration amplitude (m) |
| k | : | Constant | φ,Ψ |  | Goursat function |
| K | : | Bulk modulus (MPa) | *C* | : | Soil cohesion (MPa) |
| *K*_c_ | : | Permeability (m²) | *u_x_,u_z_* |  | Horizontal and vertical displacement (m) |
| L | : | Length of the waterfall (m) | **ISRM** |  | International Society for Rock Mechanics |
| *n*_w_ |  | Wave number | *k_a_ , k_b_*, *k_c_* | : | Proportionality constants |
| *p* | : | External pressure (MPa) | *E* | : | Modulus of elasticity (MPa) |
| *p_0_*​ | : | Internal pressure (MPa) | *e_v_* | : | Void ratio |
| Q | : | Average water flow (m³/s ) | *F_a_*​ | : | Applied force (N) |
| R/h |  | Ratio of radial distance to depth | *F_c_* | : | Compressive strength (N) |
| S | : | Storativity | *F_T_* | : | Tensile strength (MPa) |
| *u*_ε_ |  | Initial flow velocity (m/s) | G | : | Shear modulus ( MPa) |
| σ | : | Stress related to the Hooke’s law (MPa) | U | : | Settlement (m) |
| *V_s_* | : | Volume of the solid (m³) | Q | : | Average flow rate (m³/s) |
| *V_T_* | : | Total volume of water (m³) | T |  | Transmissivity (m²/day) |
| *V_V_* | : | Volume of the air-water space (m³) | *v*_f_ |  | Flow velocity (m/s) |
| w | : | Well discharge (m³/s) | μ |  | Dynamic viscosity of water (MPa·s) |
| *w*_T_ |  | Total water flow (m³/s) | *Q_fault_* | : | Quantity of faults per surface unit |
| α | : | Inclination (°) | *T^n^*(x) | : | Surface force density function (N/ m²) |
| ΔH | : | Hydraulic gradient | μ | : | Viscosity of water (MPa·s) |
| ζ | : | Overlap period (s) | T | : | Transmissivity (m²/s) |
| λ | : | Compression coefficient (MPa^−1^) | ε | : | Strain |
| *ρ* | : | Density of water (kg/m³) | Iv | : | Vibration index (m) |
| τ(x,z) |  | Shear stress (MPa) | $u'_{\varepsilon}$ | : | Propagation velocity (m/s) |
| φ | : | Internal friction angle (∘) | $\Delta p$ | : | Pressure gradient vector (MPa) |
| ω |  | Periodic frequency flow (m³/s) | *F* (t) | : | Total force (N) |
| *S_r_* |  | Cross-sectional area (m^2^) | *F_0_* | : | Initial force of magnitude (N) |
| ϕ | : | Soil porosity (%) | **ASTM** |  | American Society for Testing and Materials |
